# Pooling random forest and functional data analysis for biomedical signals supervised classification: Theory and application to electrocardiogram data

**DOI:** 10.1002/sim.9353

**Published:** 2022-02-20

**Authors:** Fabrizio Maturo, Rosanna Verde

**Affiliations:** ^1^ Department of Mathematics and Physics University of Campania Luigi Vanvitelli Caserta Italy

**Keywords:** functional between groups variability, functional between leaves variability, functional classification trees, functional data analysis, functional random forest

## Abstract

Scientific progress has contributed to creating many devices to gather vast amounts of biomedical data over time. The goal of these devices is generally to monitor people's health conditions, diagnose, and prevent patients' diseases, for example, to discover cardiovascular disorders or predict epileptic seizures. A common way of investigating these data is classification, but these instruments generate signals often characterized by high dimensionality. Learning from these data is definitely a challenging task due to many issues, for example, the trade‐off between complexity and accuracy and the course of dimensionality. This study proposes a supervised classification method based on the joint use of functional data analysis, classification trees, and random forest to deal with massive biomedical data recorded over time. For this purpose, this research suggests different original tools to extract features and train functional classifiers, interpret the classification rules, assess leaves' quality and composition, avoid the classical drawbacks due to the COD, and improve the accuracy of the functional classifiers. Focusing on ECG data as a possible example, the final purpose of this study is to offer an original approach to identify and classify patients at risk using different types of biomedical signals. The results confirm that this line of research is exciting; indeed, the interpretative tools show evidence to be very useful for understanding classification rules. Furthermore, the performance of the proposed functional classifier, in terms of accuracy, is excellent because the latter breaks the previous classification record regarding a well‐known ECG dataset.

## 1 INTRODUCTION

In recent decades, instruments to collect massive volumes of biomedical data have evolved due to technological innovations. Therefore, today, many medical devices can record different types of signals to monitor people's health conditions, diagnose, and prevent diseases. For example, electrocardiograms (ECG) are useful to track cardiac activity and diagnose a heart attack, and electroencephalograms (EEG) are helpful for brain monitoring to predict epileptic seizures. Supervised and unsupervised classification, feature extraction, and dimensionality reduction techniques are among the most used strategies for dealing with these types of data; indeed, biomedical data are often characterized by high dimensionality, and thus, appropriate methodological approaches are required. Definitely, learning from high‐dimensional biomedical data is always challenging due to the course of dimensionality (COD) that may drive to data sparsity, troubles in selecting a unique statistical model, multicollinearity, and distance concentration. In addition, other potential concerns may arise: the sampling units might be observed in a restricted set of time points that may be irregularly spaced and diverse for each statistical unit; computational time‐consuming and algorithm convergence may be complicated due to probable local minimum; the research for a trade‐off between accuracy and complexity; and finally, the interpretation of classification rules may become very hard in high‐dimensional spaces. For these reasons, in recent decades, research on classification of massive biomedical data and dimensionality reduction techniques have assumed a fundamental role in many areas, such as medicine, biostatistics, statistics, engineering, and computer science.

The starting idea of this article is that biomedical signals observed over time can be analyzed as functions in the time domain, and consequently, be treated as single objects. The latter procedure seems like the most obvious approach to deal with this type of data and, indeed, it is basically the crucial idea of functional data analysis (FDA).^1^, ^2^ The purpose of exploiting FDA to deal with biomedical data is widely shared in the literature.^3^, ^4^, ^5^, ^6^ Effectively, FDA is one of the most popular approaches to dealing with high‐dimensional data and fixing some drawbacks mentioned above, for example, COD. FDA supports reducing the dimensionality of the data and using supplementary critical sources of pattern and variation^1^ without the demand of restrictive hypotheses.^7^ For these reasons, recently, we are witnessing an uninterrupted growth of methodological research on FDA that attempts to replicate, in a functional key, a large part of classical statistics.^1^, ^2^, ^8^, ^9^, ^10^ In addition, there is a constant development of novel applications and suggestions to answer particular dilemmas in singular contexts employing functional instruments.^11^, ^12^, ^13^, ^14^, ^15^, ^16^

Because FDA is widely appreciated as a valuable tool for analyzing biomedical data, research on curves' supervised classification is lively.^7^, ^17^, ^18^, ^19^ However, functional data supervised classification using tree‐based techniques is still little known and underdeveloped. Few prior investigations dealt with such a problem and proposed very diverse approaches. Yu et al^20^ introduced spline trees for functional data, with an application to time‐of‐day patterns for consumers' international calls. Balakrishnan^21^ recommended combining functional classification trees (FCTs) and clustering. Nerini et al^22^ concentrated on the problem of regression trees to predict probability density functions via FDA. Fan et al^23^ proposed kernel‐induced random forests to predict temporal gene expression data. Gregorutti^24^ focused on variables' importance measures in tree‐based methods using FDA. Moller et al^25^ suggested extending random forest to FDA by using different mean values calculated at varying time windows as possible features. El Haouij et al^26^ recommended using functional random forest (FRF) via wavelet basis with an application to driver's stress level classification. Finally, Belli and Vantini^27^ focused on constrained convex optimization to select multiple weighted integral characteristics from the input functions and define binary splits of trees trained utilizing functional inputs. These above‐mentioned approaches are very different from each other and represent the first studies on the possible combined use of tree‐based techniques and FDA. The common idea of these studies is certainly to extend classical classification trees to a datum that can be represented through a curve. The reason is that tree‐based techniques are precise classification tools and powerful instruments that help the interpretation of classification rules; therefore, in a world of “streaming data” where high‐dimensionality has become a very common issue to deal with, pooling FDA and decision trees would lead to accurate and interpretable classifiers, dimensionality reduction, and additional features to extract information from the original data.

With the same starting idea but with a different strategy, this research intends to pool tree‐based techniques and FDA for supervised classification of curves representing high‐dimensional biomedical data recorded over time. Remarkably, this investigation proposes FCTs and FRF. Using an application on ECG data, this study shows the power of the proposed functional classifiers in terms of accuracy and their usefulness in terms of interpretability of the classification rules. Based on the technique utilized to describe the curves, various solutions are presented to decrease the dimensionality of the data and represent phenomena. In this research, the b‐spline representation and the functional principal components' decomposition (FPCD) are proposed as possible basis transformations to obtain features from curves and train FCTs and FRF classifiers. The first original part of this article is the introduction of new tools to support interpreting the functional classification rules in the functional framework, that is, the so‐called empirical splitting curve (ESC) and theoretical splitting curve (TSC). The second innovative proposal concerns the presentation of different functional measures to evaluate the variability between groups (within the same terminal node) or between terminal nodes (given the group) and their interpretation (with particular attention to the case of a binary outcome and therefore in terms of true positives, false positives, true negatives, and false negatives). The result of the joint use of these tools linked to the construction of a functional classification tree provides a powerful tool to interpret the classification rule and understand, in the time domain, how the discriminatory rule operates and how reliable it is.

This study shows compelling results from various viewpoints. First, it's worth noticing that the FRF classifier, in terms of accuracy, breaks the previous world record on a well‐known ECG dataset. Next, the concept of employing the FPCD to derive new features for training a functional classifier is a charming proposal because it enables refining the classification rules from noise and redundant information of signals in the time domain. The last aspect drives to a more steady classifier when implemented on a test set because it reduces overfitting by controlling the number of FPCs used to represent the original data. Then, the representation of the FCT through our interpretative instruments is very natural and effective in explaining the purpose of each split in a functional context. Consequently, aiming to reduce the variance of a single FCT and decorrelate FCTs in the view of a collection of FCTs, the purpose of extending random forest to FDA demonstrates to be very appealing and worthy of additional studies. In summary, this line of investigation turns out to be very promising in a world that is increasingly dominated by considerable volumes of data that we continuously gather from various devices in multiple domains of application, for example, the medical one.

This research is structured as follows. Section 2 provides an introduction to FDA, FPCD, and the most used proximity measures among functional data. Section 3 illustrates FCTs and many innovative tools to help their interpretation and assess the quality and composition of each leaf. Section 4 displays the FRF procedure. Section 5 shows a detailed application of the proposed method to ECG data with two classes to predict. Moreover, the results of another application on ECG data, with a different number of classes, is presented. A comparison between the proposed approach and some well‐known supervised classification methods for functional data is shown. Finally, Section 6 gives the conclusions and suggests possible prospective extensions of this study.

## 2 FUNCTIONAL DATA REPRESENTATION

The fundamental idea of FDA is to manage data functions as single objects. However, in practical applications, functional data are usually observed as series of point data. Thus, the function denoted by z=f(x) reduces to a record of discrete measurements that are indicated by the *T* pairs $\left(x_{j};z_{j}\right)$ where x∈ℜ and $z_{j}$ are the values of the function calculated at the points $x_{j}$, j=1,2,…,T.^1^ Generalizing the reference framework, a functional variable *X* is a random variable assuming values in a functional space Ξ, and a functional data set is a sample $x_{1},\ldots ,x_{N}$, also denoted $x_{1}(t)$ ,…, $x_{N}(t)$, drawn from a functional object.^28^

Focusing our attention to the case of a Hilbert space with a metric d(·,·) associated with a norm so that

(1) $$d(x_{1}(t),x_{2}(t))=\|x_{1}(t)-x_{2}(t)\|,$$

and where the norm ‖·‖ is associated with an inner product ⟨·,·⟩ so that

(2) $$\|x(t)\|={\left\langle x(t),x(t)\right\rangle }^{1/2},$$

we can obtain as a specific case the space ${ℒ}_{2}[a,b]$ of real square‐integrable functions defined on [a,b] by

(3) $$\langle x_{1}(t),x_{2}(t)\rangle =\int_{a}^{b}x_{1}(t)x_{2}(t)dt.$$

If $x(t)\in {ℒ}_{2}$, a basis function system is a set of known functions $\varphi_{j}(t)$ that are linearly independent of each other and which span ${ℒ}_{2}$.^1^ The first step in FDA is to convert the observed values $x_{i1},x_{i2},\ldots ,x_{iT}$ for each unit i=1,2,…,N to a functional form. The most popular method to estimate the functional datum is the basis approximation. Depending on the characteristics of the curves, various basis systems can be adopted. A simple approach is that functions can be obtained employing a finite representation in a fixed basis system^1^ as follows:

(4) $$x_{i}(t)\approx \overset{S}{\underset{s=1}{\sum }}c_{is}\varphi_{s}(t),$$

where $c_{i}$ is the vector of coefficients defining the linear combination of the *i*th curve, $\varphi_{s}(t)$ is the *s*th basis function with s∈S (*S* being a finite set of fixed basis).

A different general approach consists of employing a data‐driven basis rather than a fixed basis system. The most common procedure is the functional principal component decomposition (FPCD). The latter leads to a dimensionality reduction whilst maintaining the maximum volume of information from the initial data.^1^, ^8^, ^29^ In this situation, the functional data can be approximated as follows:

(5) $$x_{i}(t)\approx \overset{K}{\underset{k=1}{\sum }}\nu_{ik}\xi_{k}(t),$$

where *K* is the total number of selected FPCs, $\nu_{ik}$ is the score of the generic FPC $\xi_{k}$ for the generic function $x_{i}(t)$ (i=1,2,…,N). Therefore, we can achieve an approximation of the sample curves, whose explained variance is given by the sum of the eigenvalues $\sum_{k=1}^{K}\lambda_{k}$. Particularly, when dealing with high‐dimensional data, the latter dimensionality reduction technique is necessary for explaining the main features of the data by a reduced set of uncorrelated FPCs.

This approach is clearly an extension of the classical PCA. Hereafter, we quickly recall the basics of FPCD. If we assume that the observed curves are centered so that the sample mean is equal to 0, the *i*th FPCs scores are given by

(6) $$\nu_{ik}=\int_{T}x_{i}(t)\xi_{k}(t)\;dt\;\;\;i=1,\ldots ,N,$$

where the weight function $\xi_{k}$ is obtained by maximizing the variance, solving:

(7) $$Max_{f}Var[\int_{T}x(t)\xi_{k}(t)\;dt],$$

s.t.

(8) $$|\xi |{|}^{2}=\int \xi_{k}{\left(t\right)}^{2}dt=1,$$

and

(9) $$\int \xi_{k}(t)\xi_{l}(t)\;dt=0\;\;\;\;for\;\;\;\;l\neq k.$$

### 2.1 Most used proximity measures amongst functional data

Proximity measures amongst statistical units have a decisive role in FDA. Evidently, according to various chosen distances, contrasting results can be obtained. Hence, the selection of a proximity measure depends on the nature of data and aim of the analysis. In the context of the FDA, several metrics and semi‐metrics have been proposed over time; nevertheless, focusing on the case of the ${ℒ}_{2}$‐space, the most frequently used distance between curves are the following.^1^, ^2^, ^29^, ^30^ The $L_{2}$‐distance is the most utilized and can be computed as follows:

(10) $${\left\|x_{1}(t)-x_{2}(t)\right\|}_{2}={\left\{\frac{1}{\int_{a}^{b}w(t)dt}\int_{a}^{b}{\left|x_{1}(t)-x_{2}(t)\right|}^{2}w(t)dt\right\}}^{1/2},$$

where w(t) is a strictly positive weight function, and the observed points on every curve are equally spaced. Usually, the semi‐metric of the *r*‐order derivatives of two curves, for example, $x_{1}(t)$ and $x_{2}(t)$, could be considered because it produces interesting information depending on the purpose of the study. It can be calculated as follows:

(11) $$d_{2}^{\left(r\right)}\left(x_{1}(t),x_{2}(t)\right)={\left(\frac{1}{T}\int_{T}{\left(x_{1}^{\left(r\right)}(t)-x_{2}^{\left(r\right)}(t)\right)}^{2}dt\right)}^{\frac{1}{2}},$$

where $x_{1}^{\left(r\right)}(t)$ and $x_{2}^{\left(r\right)}(t)$ are the *r*‐derivatives of $x_{1}(t)$ and $x_{2}(t)$, respectively. Finally, the semi‐metric of the FPCs is especially attractive when researchers require dimensionality reduction and wish to understand similarity among curves according to distinct parts of the domain. An additional advantage is that such a measure reduces noise while keeping the most important piece of information. The semi‐metric of the FPCs is provided by:

(12) $$d_{2}\left(x_{1}(t),x_{2}(t)\right)\approx {\left(\overset{K}{\underset{k=1}{\sum }}{\left(\nu_{1k}-\nu_{2k}\right)}^{2}\left\|\xi_{k}\right\|\right)}^{\frac{1}{2}},$$

where $\nu_{i,k}$ is the score of the linear combination, and $\xi_{k}$ is the *k*th functional principal components.

## 3 FUNCTIONAL CLASSIFICATION TREES (FCTS)

The functional classification framework intends to predict an outcome *Y* by means of a variable *X* taking values in a separable metric space (Ξ,d). Theoretically, *Y* could be both categorical or numerical, leading to classification or regression problems, respectively. Nevertheless, this research concentrates on the scalar‐on‐function classification problem and thus, we consider the case of a categorical outcome *Y*. Hence, the procedure is designed for functional data of the form $\left\{y_{i},x_{i}(t)\right\}$, with a predictor curve $x_{i}(t)$, t∈T, and $y_{i}$ being the response value observed at sample i=1,…,N. Let the feasible values of Y be 0 or 1, classification of a novel observation *x* from *X* is carried out by a mapping f:F→{0,1}, called a “*classifier*,” which maps *x* into its predicted label. The latter problem can easily be extended to the case of Y with multiple modalities.

A decision tree (DT) classifier is one of the most successful supervised learning techniques to predict values of responses by learning decision rules from features.^31^, ^32^, ^33^ The starting idea is that DTs can be extended to the FDA framework by employing the scores of a fixed basis system like those in Equation (4) or (5) as original features to train the functional classifier. In the case of a data‐driven basis system, for example, Equation (5), the features' matrix is given by:

(13) $$V=\left(\begin{array}{ccc} \nu_{11} & \ldots & \nu_{1K}\\ \vdots & \ddots & \vdots \\ \nu_{N1} & \ldots & \nu_{NK} \end{array}\right),$$

where $\nu_{ik}$ is the score of the *i*th curve (i=1,…,N) relative to the *k*th functional principal component $\xi_{k}$ (k=1,…,K).

In the instance of a fixed basis system, for example, Equation (4) using B‐splines, the features' matrix is given by:

(14) $$C=\left(\begin{array}{ccc} c_{11} & \ldots & c_{1S}\\ \vdots & \ddots & \vdots \\ c_{N1} & \ldots & c_{NS} \end{array}\right),$$

where the generic element $c_{is}$ is the coefficient of the *i*th curve (i=1,…,N) relative to the *s*th (s=1,…,S) basis function $\varphi_{s}(t)$ involved in the linear combination. The most challenging element of these strategies is to look for a functional interpretation of the classification rules provided by FCTs. For this reason, the selection of one of the two approaches depends on diverse reasoning, for example, desired interpretability and dimensionality reduction needs.

A FCT consists of recursive binary separations of the feature space into rectangular regions (terminal nodes or leaves) made by collections of curves $x_{i}(t)\in X$. To build the FCT, an optimal binary separation is provided at every step of the algorithm, based on the optimization of a cost criterion (eg, decrease of the impurity of the node via the Gini or Shannon‐Weiner index).^33^, ^34^

The Gini index is a measure of heterogeneity for categorical variables, and thus the lower the value of the index, the more homogeneous the observations in a node (the more the node is “pure”). It can be computed as follows:

(15) $$G=1-\overset{F}{\underset{i}{\sum }}f_{zi}^{2},$$

where $f_{zi}$ is the proportion of training observations in the *z*th node that are from the *i*th class, and *F* is the number of modalities of the outcome *Y*.

The Shannon‐Wiener entropy index is also an index of heterogeneity for categorical variables, and thus the interpretation is similar to the Gini's index. It can be calculated as follows:

(16) $$E=\overset{F}{\underset{i}{\sum }}f_{zi}\cdot \log f_{zi},$$

where $f_{zi}$ represents the proportion of training observations in the *z*th node that are from the *i*th class.^33^, ^35^

The algorithm starts with the entire functional data set using the scores of the FPCD obtained using Equation (13) or the coefficients obtained using Equation (4), and proceeds until the leaves are obtained. Having obtained the best split in one node, the data are partitioned into two nodes; the rule is replicated to produce the most proper binary division on all resulting nodes. Typically, a vast FCT is produced at the beginning, which is then pruned according to an optimization criterion, for example, to look for an acceptable trade‐off between complexity and accuracy. In summary, the FTC is an extension of the classical classification tree algorithm (see eg, References 35, 36, 37) to the functional context.

### 3.1 The theoretical splitting curve (TSC) and empirical splitting curve (ESC)

Given that the scores of the linear combination are used as new features to predict the response Y, the interpretation of FCT is totally different if compared to the classical CTs. Indeed, the values of the splits should be interpreted according to the part of the domain that the single FPC $\xi_{k}(t)$ or B‐Spline $\varphi_{s}(t)$, mostly describe and the scores' thresholds $\nu_{0k}$ or $c_{0s}$, respectively. The subscript “0” (rather than *i*) in $\nu_{0k}$ and $c_{0s}$ symbolizes that the threshold recognized for the score relating to a specific FPC $\xi_{k}(t)$ or B‐spline $\varphi_{s}(t)$ becomes a fixed value to divide the curves into two subsamples (son nodes). Effectively, $\nu_{0k}$ and $c_{0s}$ are fixed value that does not depend on the *i*th unit but are the same for all the units. For example, contemplating the first split rule on a hypothetical FCT based on FPCs, that is, the division rule of the root node, $\nu_{0k_{0}}$ is the threshold value related to a generic FPC $K_{0}$. Thus, all the curves meeting the condition $\nu_{ik_{0}}<\nu_{0k_{0}}$ form a subgroup whereas all the residual functions, that is, those meeting the condition $\nu_{ik_{0}}\geq \nu_{0k_{0}}$, enter the other subset.

To make the split rule better readable in the FDA setting, we offer two notions: the TSC and ESC. The TSCs are given by each theoretical separation rules generated by every single split of the FCTs. In the case of a data‐driven basis system, for example, Equation (5), the TSP can is given by:

(17) $$TSC_{z}^{FPC}(t)=\underset{k\in R}{\sum }\nu_{0k}\xi_{k}(t),$$

where *R* is the set of FPCs $\xi_{k}(t)$ involved in the classification rule path until the splitting of the *z*th intermediate node (z=1,…,Z). Therefore, the generic intermediate node that produces a cut is indicated with z, and the whole number of these middle nodes is identified with *Z*. Consequently, each $TSC_{z}^{FPC}(t)$ can be associated to each intermediate node and, of course, also to the root node ($TSC_{1}^{FPC}(t)$). The limit of $TSC_{z}^{FPC}(t)$ is that it does not help to understand the separating laws in terms of the time domain. Indeed, TSCs are generally very smooth with few functional variability.

Consequently, we introduce the ESC. The latter is represented by the curve, living in the training dataset, which is the closest (based on the semi‐metric FPCs) to the separation rule given by $TSC_{z}^{FPC}(t)$ and can be defined as follows:

(18) $$ESC_{z}^{FPC}(t)=x^{(z)FPC}(t)=\underset{i}{\text{argmin}}\;\;d_{2}\left(x_{i}(t),TSC_{z}^{FPC}(t)\right).$$

Henceforth, $ESC_{z}^{FPC}(t)$ is a curve outlining the functional empirical separation rule provided by the binary separation of a node split and is very helpful to understand the separation rule over the time domain. It follows that we can identify and plot a $ESC_{z}^{FPC}(t)$ for every split *z* being in the FCT based on FPCs. Therefore, we can always identify a $ESC_{z}^{FPC}(t)$ associated to each $TSC_{z}^{FPC}(t)$. This strategy serves in explaining the various levels of separation in the FCT based on FPCs.

In the instance of a fixed basis system, for example, Equation (4) using B‐splines, the TSP is given by:

(19) $$TSC_{z}^{BSP}(t)=\underset{s\in Q}{\sum }c_{0s}\varphi_{s}(t),$$

where *Q* is the number of b‐splines $\varphi_{s}(t)$ used in the classification rule path until the split of the *z*th node (z=1,…,Z).

Instead, the TSC can be defined as follows:

(20) $$ESC_{z}^{BSP}(t)=x^{(z)BSP}(t)=\underset{i}{\text{argmin}}\;\;{\left\|x_{i}(t)-TSC_{z}^{BSP}(t)\right\|}_{2}$$

Hence, the distance used to look for $ESC_{z}^{BSP}(t)$ is provided by Equation (10) whereas the semi‐metric adopted to identify $ESC_{z}^{FPC}(t)$ is given by Equation (12).

### 3.2 Leaves quality assessment using functional tools

After pruning the FCT to look for an optimal trade‐off between complexity and accuracy, the final nodes are composed of curves belonging to different classes. Therefore, using the Gini or Shannon‐Weiner indexes^33^, ^35^ (see Equations (15) and (16)), it is straightforward to observe that a pruned FCT is often made of many terminal nodes that are not pure. In a classical non‐functional classification tree, it is common to refer only to the classical measures of heterogeneity above‐mentioned to check for the impurity of a terminal node. However, in the context of FDA and FCTs, it is possible to exploit supplementary sources of information furnished by the functional nature of the data in each node. For this reason, in this subsection, many additional criteria to evaluate the quality of the leaves in terms of variability are presented.

#### 3.2.1 The functional variability of a leaf

The functional variability of the leaves is introduced as extra information about the characteristics of the decision rules. In other words, the variability of the terminal nodes can be computed according to two different types of measures, and thus we can get a double source of information. The first source of variability is given by the heterogeneity computed using the Gini or the Shannon‐Weiner index; therefore, we have two measures of the impurity of a node determined according to the distribution of the curves based on the different modalities of the outcome Y. From a conceptual and also practical perspective, the only difference with respect to the classical case is that individuals are represented by curves belonging to different classes rather than scalar values. The second source of variability is given by the functional dispersion of the curves in each leaf around the functional mean of the same leaf. In other words, despite the curves in each leaf can belong to different classes, we can also measure the functional deviance that examines the leaf variability over the whole reference domain. The functional deviance of a leaf is given by:

(21) $$DEV^{\left(l\right)}(t)=\overset{n^{\left(l\right)}}{\underset{i=1}{\sum }}(x_{i}(t)-{\bar{x}}^{\left(l\right)}(t){)}^{2}\;\;\;\;\;i\in l,$$

where $DEV^{\left(l\right)}(t)$ is the functional deviance of the generic *l*th leaf, $n^{\left(l\right)}$ is the number of curves in the *l*th leaf (l=1,…,L), ${\bar{x}}^{\left(l\right)}(t)$ is the functional mean of the *l*th leaf. Large values of $DEV^{\left(l\right)}(t)$ furnishes information on time intervals in which the curves belonging to the same terminal node are very far from the functional mean of the same leaf, and therefore the curves are diverse from each other. In other words, the latter would be evidence of a terminal node composed of different functions, regardless of the labels of the response variable *Y* that do not contribute to the calculation of this type of variability. Conversely, a moderate value of $DEV^{\left(l\right)}(t)$ symbolizes a leaf with very similar curves.

To display the functional variability of the terminal nodes, in terms of the total functional deviance of the root node, $DEV^{\left(l\right)}(t)$ can be depurated by the total variability as follows:

(22) $${}_{rel}DEV^{\left(l\right)}(t)=\frac{\sum_{i=1}^{n^{\left(l\right)}}(x_{i}(t)-{\bar{x}}^{\left(l\right)}(t){)}^{2}}{\sum_{i=1}^{N}(x_{i}(t)-\bar{x}(t){)}^{2}}\;\;\;\;\;i\in l,$$

where $\sum_{i=1}^{N}(x_{i}(t)-\bar{x}(t){)}^{2}$ is the functional deviance of the root node and $\bar{x}(t)$ is its functional mean. The latter tool can be useful to compare the quality of the leaves and better understand their composition according to different parts of the time domain.

#### 3.2.2 The functional between groups variability within a single leaf

Based on the classification rule of a FCT, the perfect result would consist of getting all the curves in a terminal node with the same predicted class. Nevertheless, in a pruned FCT, the nodes are usually impure, and thus there are some leaves composed of incorrectly classified curves. To capture the variability among different groups in a leaf, the functional between groups sum of squares (FBGSS) of the leaf can be used, the latter can be defined as follows:

(23) $$FBGSS^{\left(l\right)}(t)=\overset{G}{\underset{g=1}{\sum }}n_{g}^{\left(l\right)}{\left({\bar{x}}_{g}^{\left(l\right)}(t)-{\bar{x}}^{\left(l\right)}(t)\right)}^{2},$$

where *G* is the total number of groups, $n_{g}^{\left(l\right)}$ is the number of curves of the *g*th group in the *l*th leaf, ${\bar{x}}^{\left(l\right)}(t)$ is the total functional mean in the *l*th terminal node, and

(24) $${\bar{x}}_{g}^{\left(l\right)}(t)=\overset{n_{g}^{\left(l\right)}}{\underset{i=1}{\sum }}\frac{x_{i}(t)}{n_{g}^{\left(l\right)}}\;\;\;\;\;i\in l,$$

is the functional mean of the *g*th group in the leaf *l*.

To obtain a more refined functional measure, depurated by the variability of the terminal node *l*, $FBGSS^{\left(l\right)}(t)$ can be divided by the total variance of the leaf as follows:

(25) $${}_{rel}FBGSS^{\left(l\right)}(t)=\frac{FBGSS^{\left(l\right)}(t)}{DEV^{\left(l\right)}(t)}.$$

##### 3.2.2.1 **A particular interpretation of the functional between groups variability within a single leaf when the response variable is binary**

Let the possible values of Y be 0 (“*Disease*”) or 1 (“*Healthy*”) and a classification problem where the aim is to predict the presence/absence of a specific disease in people at risk. It follows that the predicted classes for new curves in the test set can be “*Positive*” or “*Negative*” depending on the classification rule of the FCT, and thus, on the terminal node where the patient will be attributed. As in the classic non‐functional case, if the class predicted by the leaf is “*Disease*” and the patient is ill, then there is a true positive (TP). If the classification rule makes a mistake because the patient is healthy, there is a false positive (FP). If the class predicted in the leaf is “*Healthy*,” and the patient is healthy, then there is evidence of a true negative (TN). Conversely, if the patient is sick, a false negative (FN) is present. Starting from the above described classical approach to define FPs, FNs, TPs, and TNs, ad‐hoc functional measures to get extra information on leaves' quality can follow a specific notation.

When the predicted class of a terminal node *l* is “*Disease*,” the functional mean of the TP curves in the leaf *l* can be indicated as follows:

(26) $${\bar{x}}_{TP}^{\left(l\right)}(t)=\overset{n_{TP}^{\left(l\right)}}{\underset{i=1}{\sum }}\frac{x_{i}(t)}{n_{TP}^{\left(l\right)}}\;\;\;\;\;i\in l,$$

where $n_{TP}^{\left(l\right)}$ is the total number of TP curves in the leaf *l* with $n_{TP}^{\left(l\right)}\leq n^{\left(l\right)}$. Instead, the functional mean of the FP functions in the leaf *l* is given by:

(27) $${\bar{x}}_{FP}^{\left(l\right)}(t)=\overset{n_{FP}^{\left(l\right)}}{\underset{i=1}{\sum }}\frac{x_{i}(t)}{n_{FP}^{\left(l\right)}}\;\;\;\;\;i\in l,$$

where $n_{FP}^{\left(l\right)}$ is the total number of FP functions in the leaf *l* with $n_{FP}^{\left(l\right)}\leq n^{\left(l\right)}$, and $n^{\left(l\right)}=n_{TP}^{\left(l\right)}+n_{FP}^{\left(l\right)}$.

$FBGSS^{\left(l\right)}(t)$ and ${}_{rel}FBGSS^{\left(l\right)}(t)$, in this context, are indicated with $FBGSS_{TP-FP}^{\left(l\right)}(t)$ and ${}_{rel}FBGSS_{TP-FP}^{\left(l\right)}(t)$, respectively, to stress that the focus is on a leaf predicting a “*Disease*” status. The assessment and plot of ${}_{rel}FBGSS_{TP-FP}^{\left(l\right)}(t)$ across the entire domain provides interesting information about the composition of the leaf and why TP and FP curves are in the same terminal node *l*. Effectively, given the variance of the node, the lower ${}_{rel}FBGSS_{TP-FP}^{\left(l\right)}(t)$, the more similar ${\bar{x}}_{TP}^{\left(l\right)}(t)$ and ${\bar{x}}_{FP}^{\left(l\right)}(t)$; thus, despite the actual health condition of some patients, they are badly classified because their signals are similar to diseased people in certain parts of the time domain. Evidently, the parts of the domain in which these similarities exist resulted in the same predicted class. Instead, the higher ${}_{rel}FBGSS_{TP-FP}^{\left(l\right)}(t)$, the greater the difference between ${\bar{x}}_{TP}^{\left(l\right)}(t)$ and ${\bar{x}}_{FP}^{\left(l\right)}(t)$ in some parts of the time domain. In the latter instance, there is an important indication of where the actual functional difference between TPs and FPs is. In other words, it is possible to identify those characteristics of the FPs' signals that has been ignored by the classification rule, resulting in a classification error. As an extreme case, ${}_{rel}FBGSS_{TP-FP}^{\left(l\right)}(t)=0$ may happen even if it is quite unlikely but still possible. In fact, in the presence of an impure node and equality between ${\bar{x}}_{TP}^{\left(l\right)}(t)$ and ${\bar{x}}_{FP}^{\left(l\right)}(t)$ over the whole domain, we have a situation in which it is almost impossible to train a functional classifier that would be precise for those statistical units. In fact, it would mean that identical curves have different original labels. Training a classifier with good chances to correctly predict the class of those similar functions would be quite impossible because the latter share the same features.

Following the above reasoning, the same idea can be extended to the case in which the predicted class of a terminal node *l* of a FCT is “*Healthy*.” The functional mean of the TN curves in the leaf *l* is given by:

(28) $${\bar{x}}_{TN}^{\left(l\right)}(t)=\overset{n_{TN}^{\left(l\right)}}{\underset{i=1}{\sum }}\frac{x_{i}(t)}{n_{TN}^{\left(l\right)}}\;\;\;\;\;i\in l,$$

where $n_{TN}^{\left(l\right)}$ is the total number of TN curves in the leaf *l* with $n_{TN}^{\left(l\right)}\leq n^{\left(l\right)}$. Instead, the functional mean of the FN functions in the leaf *l* is given by:

(29) $${\bar{x}}_{FN}^{\left(l\right)}(t)=\overset{n_{FN}^{\left(l\right)}}{\underset{i=1}{\sum }}\frac{x_{i}(t)}{n_{FN}^{\left(l\right)}}\;\;\;\;\;i\in l,$$

where $n_{FN}^{\left(l\right)}$ is the total number of FN functions in the leaf *l* with $n_{FN}^{\left(l\right)}\leq n^{\left(l\right)}$, and $n^{\left(l\right)}=n_{TN}^{\left(l\right)}+n_{FN}^{\left(l\right)}$.

The same reasoning described for TPs and FPs can be extended to the case of TNs and FNs. $FBGSS^{\left(l\right)}(t)$ and ${}_{rel}FBGSS^{\left(l\right)}(t)$, in this circumstance, are indicated with $FBGSS_{TN-FN}^{\left(l\right)}(t)$ and ${}_{rel}FBGSS_{TN-FN}^{\left(l\right)}(t)$, respectively, to remark we are focusing on a leaf predicting a “*Healthy*” status. In this case, high values of ${}_{rel}FBGSS_{TN-FN}^{\left(l\right)}(t)$ indicate that despite TNs and FNs have different behaviors in some parts of the time domain, they share the same predicted class. Analyzing the intervals where this difference is most marked is a clue to detect those functional features that have been neglected by the classification rule and led to the increase of the final misclassification error of the functional classifier.

In this section, there is no distinction according to the basis system. Indeed, the proposed strategy can be adopted whatever the base system used because the former is based on leaves' composition rather than how curves are represented.

#### 3.2.3 The functional between leaves variability of all the leaves for a single group

In the previous section, in addition to the classical impurity measures, some original procedures to evaluate the quality of a specific leaf based on the between groups variability in that specific leaf are introduced. Thus, in the latter approaches, only individual terminal nodes and their composition are taken into account. Nevertheless, common questions that would arise in this peculiar context are the following: what is the behavior of the functional mean of a subgroup of FNs in a leaf dominated by TNs (thus leading to the predicted class “*Healthy*”) if compared to the trend of the functional mean of a subgroup of TPs in an impure leaf dominated by TPs (which therefore leads to the predicted class “*Disease*”)? And what is the behavior of the functional mean of a subgroup of FPs in an impure leaf dominated by TPs (which therefore leads to the predicted class “*Disease*”) if compared to the functional mean of a subgroup of TNs in a leaf dominated by TNs (which then leads to the predicted “*Healthy*” class)? It is possible to answer these questions by introducing the concepts of functional between leaves variability of all the leaves and functional between leaves variability of two leaves. In the following, we propose two different measures: the first one is the functional between leaves variability of one group among all the leaves; and the second one is the functional between leaves variability of a couple of leaves. The latter is a particular case of the first one, that is, when considering only two leaves and one of them is the so‐called “*reference*” leaf. The next section is dedicated to the latter functional measure.

The functional between leaves sum of squares (FBLSS) among all the leaves can be defined as follows:

(30) $$FBLSS_{g}(t)=\overset{L}{\underset{l=1}{\sum }}n_{g}^{\left(l\right)}{\left({\bar{x}}_{g}^{\left(l\right)}(t)-{\bar{x}}_{g}(t)\right)}^{2},$$

where ${\bar{x}}_{g}(t)$ is the overall functional mean for the group *g*, that is, considering all the leaves. In other words, $FBLSS_{g}(t)$ takes into account only one group *g* to detect its variability among different leaves. Consequently, it is a valuable measure to understand how leaves lead to different prototypes of the same original group *g*.

##### 3.2.3.1 **A particular interpretation of the functional between leaves variability of all the leaves for a single group when the response variable is binary**

Limiting the attention to the case of a binary outcome *Y* that can only take values (“*Disease*”) or (“*Healthy*”), $FBLSS_{TP-FN}(t)$ indicates the FBLSS taking into account all the diseased patients present in all the leaves, that is, TPs and FNs. On the other hand, $FBLSS_{TN-FP}(t)$ is the FBLSS taking into account all the healthy patients present in all the leaves, that is, TNs and FPs.

#### 3.2.4 The functional between leaves variability of two leaves for a single group

A special case of $FBLSS_{g}(t)$ can be obtained by considering only a couple of leaves instead of all the terminal nodes. Particularly, we consider arranging in descending order the terminal nodes based on the relative frequency of correct predictions available in each of them. Let the best leaf in the ranking be indicated as $l_{*}$. As stressed before, in a pruned FCT, the nodes are often impure and therefore rarely lead to a prediction that is “error‐free.” Suppose the *l*th terminal node leads to the ĝ predicted class. Hence, the *l*th terminal node has a majority of statistical units with original labels equal to *g* and a minority with an original class different from *g* (note that, if not, the overall prediction of the terminal node would be different from *g*). Using the previous example of diseased and healthy people, one would question which is the divergence in trends between diseased patients who are badly classified as “*Healthy*” and a reference curve representing diseased people who are rightly classified as “*Diseased*.” In other words, which is the difference between FNs in a specific leaf and a reference curve of TPs?

The generalized version of the FBLSS of two leaves *l* and $l_{*}$ can be given by:

(31) $$FBLSS_{g}^{\left(l)-(l_{*}\right)}(t)=n_{g}^{\left(l\right)}{\left({\bar{x}}_{g}^{\left(l\right)}(t)-{\bar{x}}_{g}^{*}(t)\right)}^{2}+n_{g}^{\left(l_{*}\right)}{\left({\bar{x}}_{g}^{\left(l_{*}\right)}(t)-{\bar{x}}_{g}^{*}(t)\right)}^{2},$$

where ${\bar{x}}_{g}^{*}(t)$ is the functional mean computed considering only the leaves *l* and $l_{*}$, $n_{g}^{\left(l_{*}\right)}$ is the total number of curves belonging to the group *g* in the reference leaf $l_{*}$. In other words, $FBLSS_{g}^{\left(l)-(l_{*}\right)}(t)$ considers only one group *g* in a specific leaf *l* with respect to a reference leaf $l_{*}$. Hence, it is a useful measure to compare two leaves leading to two different prototypes of the same original group *g*.

To obtain a more accurate functional comparison between ${\bar{x}}_{g}^{\left(l\right)}(t)$ and ${\bar{x}}_{g}^{l_{*}}(t)$, we consider a functional variability measure that is depurated by the total functional between leaves variability of the group *g* (see Equation (30)) as follows:

(32) $${}_{rel}FBLSS_{g}^{\left(l)-(l_{*}\right)}(t)=\frac{FBLSS_{g}^{\left(l)-(l_{*}\right)}(t)}{FBLSS_{g}(t)}.$$

In summary, ${}_{rel}FBLSS_{g}^{\left(l)-(l_{*}\right)}(t)$ is the proposed “*gold‐standard*” functional measure to provide a functional comparison between two groups of functions belonging to the same class *g* but in two different terminal nodes relative to the total variability of the curves belonging to the same class *g* between all the leaves.

##### 3.2.4.1 **A particular interpretation of the functional between leaves variability of two leaves for a single group when the response variable is binary**

Focusing the attention to the case of a binary outcome with possible values (“*Disease*”) and (“*Healthy*”), we can consider two different cases that help the understanding of the proposed approach. First, when focusing on diseased patients, we are interested in comparing TPs who are correctly classified in a leaf and FNs that are incorrectly classified in another leaf. Both subgroups are composed by diseased people, and thus the most interesting aspect is to capture the similarities and dissimilarities of these subgroups. Hence, the functional between leaves mean squares to compare TPs in the reference leaf and FNs in a generic leaf *l*, relative to the total between variability among all diseased patient in different leaves, is indicated as:

(33) $${}_{rel}FBLSS_{FN-TP}^{\left(l)-(l_{TP}\right)}(t)=\frac{FBLSS_{FN-TP}^{\left(l)-(l_{TP}\right)}(t)}{FBLSS_{FN-TP}(t)},$$

where the subscript “FN−TP” instead of “*g*” highlights that the subgroups of diseased patients are considered. Following the previous notation, in Equation (33), $l_{TP}$ is the reference leaf for diseased people, that is, the leaf with the most accurate prediction of disease. However, the reference leaf can also be changed according to different reasoning and ranking, for example, looking for a trade‐off between the total number of curves in the leaf and its accuracy in predicting the disease status. Evidently, the plot of ${}_{rel}FBLSS_{FN-TP}^{\left(l)-(l_{TP}\right)}(t)$ over the whole time domain provides fascinating information about the FN curves in the generic leaf *l*. Indeed, now, it is possible to answer to the first out of the two above‐mentioned questions. High values of the functional between variability over some intervals of the time domain can help to explain why FNs are in leaf *l* leading to a classification error, instead of being in the terminal node $l_{TP}$ with the other TPs. In other words, we are able to obtain a functional reason for FNs to be “*false*” instead of being “*true*” positives.

On the contrary, if the interest is on healthy patients, we aim to compare TNs who are correctly classified and FP that are incorrectly classified. Both subgroups are composed by healthy people, and thus we aim to detect similarities and dissimilarities of these subgroups. Hence, the FBLSS to compare TNs in the reference leaf and FPs in a generic leaf *l*, relative to the total between variability among all healthy patient in different leaves, is given by:

(34) $${}_{rel}FBLSS_{FP-TN}^{\left(l)-(l_{TN}\right)}(t)=\frac{FBLSS_{FP-TN}^{\left(l)-(l_{TN}\right)}(t)}{FBLSS_{FP-TN}(t)},$$

where the subscript “*FP‐TN*” instead of “*g*” highlights that all the subgroups of healthy patients are considered, and $l_{TN}$ is the reference leaf for healthy people, that is, the leaf with the most accurate prediction of health condition. Evidently, ${}_{rel}FBLSS_{FP-TN}^{\left(l)-(l_{TN}\right)}(t)$ allows us to answer the second question mentioned above, that is, explaining the difference, over the whole domain, between FPs in a leaf and TNs in a reference terminal node describing the most reliable classification rule of the FCT to predict the healthy status. High values of ${}_{rel}FBLSS_{FP-TN}^{\left(l)-(l_{TN}\right)}(t)$ allows us to explain why healthy patients ended up in the wrong path of the FCT. In other word, we can capture the functional differences between the best prototype of TNs and the specific average curve describing FPs in another leaf.

The concept of functional between leaves variability of two different leaves can be extended to any couple of nodes *l* and $j\neq l_{*}$; however, the most interesting mean comparison, in our opinion, in that one with respect to the most accurate leaf in predicting the specific class.

### 3.3 Assessing the performance of FCTs by projecting test set curves onto the FPCs space generated by the training set

To evaluate the performance of the FCT, in terms of accuracy, many possibilities exist, as in the case of the classical supervised classification. Focusing on the training set, we can consider bootstrap or cross‐validation to test the misclassification error as in the classical framework. Instead. using a test set is the most reasonable approach, but it deserves attention.

When a fixed basis system is adopted, for example, b‐splines, to represent the curves of the training set, the order and number of the b‐splines are selected. If the same order and number of basis are used also to describe the curves in the test set, the use of the splitting rules given by the FCT is straightforward. Indeed, the coefficients of the linear combination representing the functions of the test set are associated with the same basis functions used for the coefficients of the linear combination representing the functions of the training set. Instead, when a data‐driven basis system is used, for example, FPCs, computing once again FPCs but on the test set will produce a different basis system because it strictly depends on the data. Hence, the test set curves must be projected into the space spanned by the FPCs estimated on the training data. In other words, the values of the scores of the test set curves must be calculated. Using the latter strategy, the *j*th principal component score can be computed as follows:

(35) $$\nu_{ik}=\langle x_{c},\xi_{k}\rangle =\int_{T}x_{i}(t)\xi_{k}(t)\;dt\;\;\;i=1,\ldots ,M,$$

where the weight functions $\xi^{\prime }s$ are obtained performing the FPCs on the training set, $x_{c}$ are the centered functions of the test set, and *M* is the total number of curves in the test set. After expressing the curves in the test set as a function of the FPCs system estimated on the training set, then we can apply the classification rule provided by the FCT and estimate the misclassification error and accuracy of the functional classifier.

## 4 FROM FCTS TO THE ENSEMBLE: THE FUNCTIONAL RANDOM FOREST (FRF) APPROACH

Today, RF^33^, ^34^, ^38^ is recognized as one of the most efficient supervised classification algorithm. Specifically, RF is a particular case of bagging for classification trees.^37^ This study suggests extending the classical RF algorithm^39^, ^40^ to functional data with the equivalent motivations used to support the introduction of this approach in the non‐functional framework, that is, improving the performance and decreasing the variance of a single FCT. To introduce the so‐called FRF, the functional bagging procedure (FBG), that is, functional bootstrap aggregation, should be introduced in advance. The main reason to introduce FBG is that, in a single FCT, modest variations in the data may lead to very diverse FCTs and thus different classification rules. Accordingly, the starting idea of FBG is to train an ensemble of FCTs for building a terminal classification rule and reducing the variance of a single FCT.

Let FBG consists of *H* FTCs $\tau_{h}$, h=1,…,H, where *H* is chosen to be a large number. The *h*th tree $\tau_{h}$ is grown on a random subset of the training set, obtained from the original data $D=\{(y_{i},x_{i}(t)),i=1,\ldots ,N\}$ by drawing, with replacement, a bootstrap sample $D_{h}^{*}=\{(y_{s}^{\left(h\right)},x_{s}^{\left(h\right)}(t)),s=1,\ldots ,N\}$ of the same size *N* as the original data set. Thus, the set of curves i=1,…,N present in the *h*th bootstrap sample $D_{h}^{*}$ is called, from now on, “*In‐Bag Functional Data*” sample (IBFD) and is used to build the single *h*th FCT. Instead, the “*Out‐of‐Bag Functional Data*” sample (from now on, OOBFD) is composed of the remaining curves relative to those functions that are not present in $D_{h}^{*}$. Then, we train *H* FCTs using *H* bootstrapped functional training sets to get ${\hat{f}}_{h}^{*}$. Afterwards, we average all the predictions to obtain the final prediction for the *i*th curve as follows:

(36) $${\hat{f}}_{H}(x_{i}(t))=\frac{1}{H}\overset{H}{\underset{h=1}{\sum }}{\hat{f}}_{h}^{*}(x_{i}^{\left(h\right)}(t)).$$

In other words, for a given $x_{i}(t)\in D$, we register the class predicted by each of the *H* FCT, and use the so‐called “majority vote” see for example, Reference 34. The misclassification error rate can be computed as the average of the error rate of each ${\hat{f}}_{h}^{*}$. Therefore, the overall forecast for each new curve *i*th is the most usually occurring class amongst the *H* predictions according to the *H* different FCT.

Each FCT is grown deep and is not pruned, and thus each FCT has low bias, but a large variance. Hence, the advantage of FBG is that averaging these *H* FCTs decreases the variance. This procedure gains in accuracy with respect to a single FCT because it combines many FCTs. Increasing *H* will not lead to overfitting. In practice, we would use a value of *H* that is large enough for the test error to have settled down. Nevertheless, the limit of FBG is that the decrease in variance is limited because the FCTs are not independent. Effectively, it is trivial to observe that, in real applications, the FCTs are very correlated because almost all are dominated by the same features, that is, FPCs or b‐splines in our context. In practice, in most FCTs, the first separations at the top of FDTs are always dictated by the same features that can better discriminate the classes of the outcome Y.

FRF improves FBG by way of a minor tweak that decorrelates the FCTs and reduces the variance when we average many FCTs. Each time a split in an individual FCT is considered, a random selection of *m* features is chosen as split candidates from the full set formed by *K*FPCs (see Equation (13)) (or *S* b‐splines if we use Equation (14)). It follows that, when m<K (or m<S in a fixed basis system), we have FRF whereas when m=K (or m=S in a fixed basis system), we have that FBG = FRF. Following this strategy, the FCTs into the forest will be less correlated because the essential FPCs (or b‐splines) will not always be those features on the top of the FCT, determining the first significant cut rules. Focusing on FPCs, for example, a general rule of thumb can be to select, as the size of the subset of FPCs, a value of $m\approx \sqrt{K}$. Consequently, at each split in the FPC, the algorithm is not even allowed to consider most of the available features. Surely, on average, $\frac{m-K}{m}$ of the splits will not even contemplate some FPCs. In this way, FRF decorrelates the FCT, making the average of the FCT less variable and hence more reliable. Thus, the discrepancy between FBG and FRF depends on the selection of *m*.

Regarding the estimation of the misclassification error rate, in addition to using the training set, test set, or the classical cross‐validation approaches, it is also possible to exploit the OOBFD as described in Equation (36). As discussed above, the latter method has the advantage of allowing all curves in the dataset to be used to train the functional classifier. In this way, it is possible to limit the loss of information due to a possible division of the starting dataset into a training and test set. However, the accuracy of the classifier would always be slightly overestimated if compared to the use of a “pure” test set.

## 5 APPLICATIONS TO ECG DATASETS

*Cardiovascular disease* (CVD) (often used interchangeably with the term “*Hearth disease*”) is one of the most significant determinants of morbidity and death among worldwide people. For this reason, the prediction of cardiovascular disorders is one of the most critical problems in medicine and biostatistics. Undoubtedly, identifying in advance patients affected by heart disease can prevent serious consequences, for example, stroke and heart attack. One of the most used techniques to monitor heart health is the ECG which provides a representation of the electrical activity of the heart muscle over time.

This section presents the results of two applications of FCT and FRF to ECG datasets and some comparisons with different functional classifiers. In the first application, the whole method in detail is illustrated while, in the second application, only results and comparisons are presented. Specifically, our approach is applied to two well‐known datasets available at https://www.timeseriesclassification.com/: “*ECG200*” and “*ECG5000*.”

### 5.1 An application to the ECG200 dataset with a detailed description of the strategy

The ECG200 dataset was proposed by R. Olszewski at Carnegie Mellon University in 2001, as part of his work “*Generalized feature extraction for structural pattern recognition in time‐series data*.”^41^ The ECG200 dataset is continuously used to test new classifiers, and the current world record, in terms of classification accuracy, has been reached using the BOSS algorithm and is equal to 89.05%. Each series traces the electrical activity registered during one heartbeat. The two classes are normal heartbeat (NH) and myocardial infarction (MI). The data are composed of one‐hundred signals in the training set and one‐hundred in the test set. The data is freely available on the website https://www.timeseriesclassification.com/description.php?Dataset=ECG200.^42^ Our goal is to predict whether a new patient, whose ECG we observe, is healthy or diseased.

**FIGURE 1:**
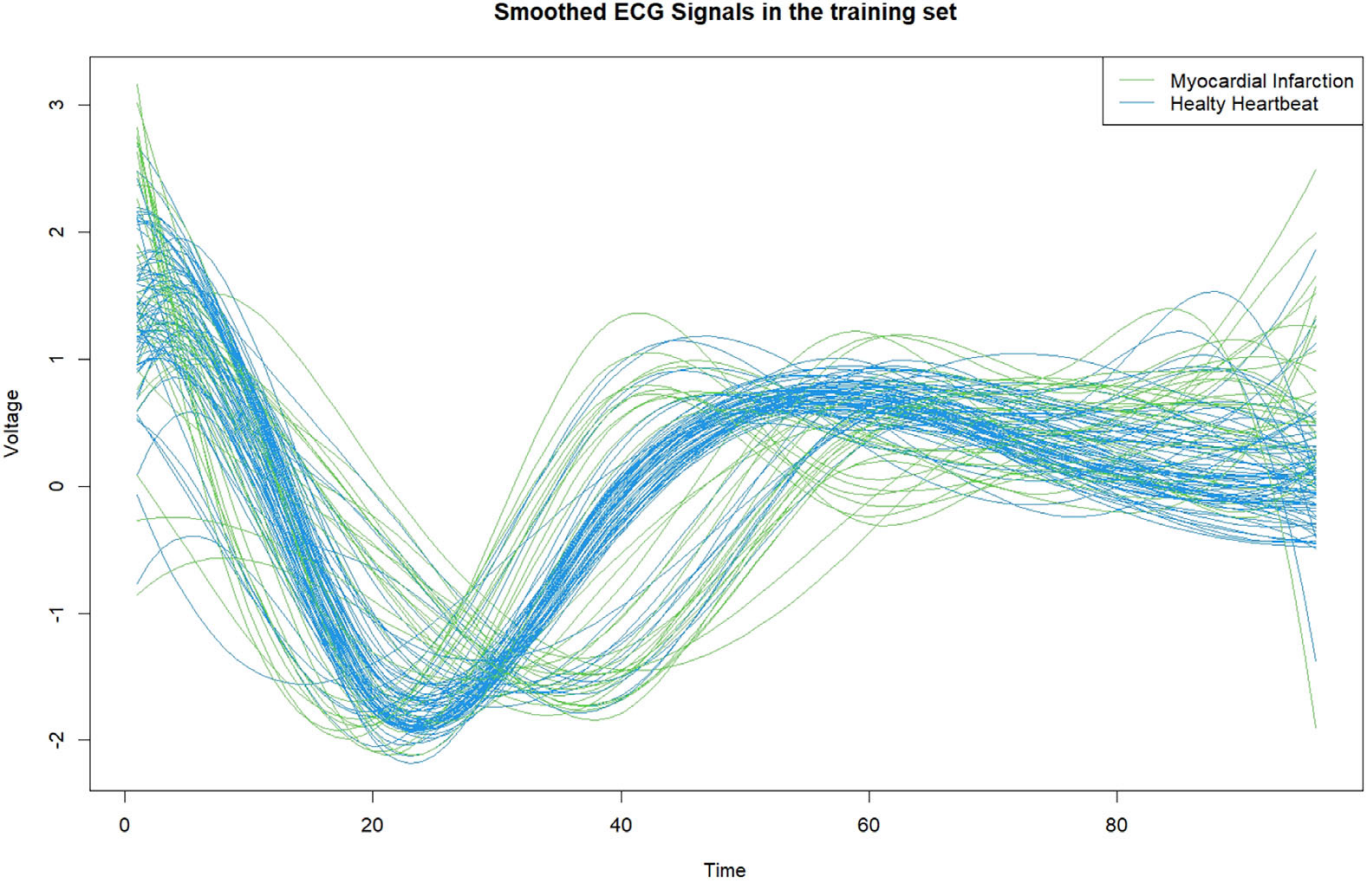
Smoothed ECG functions in the training dataset

**FIGURE 2:**
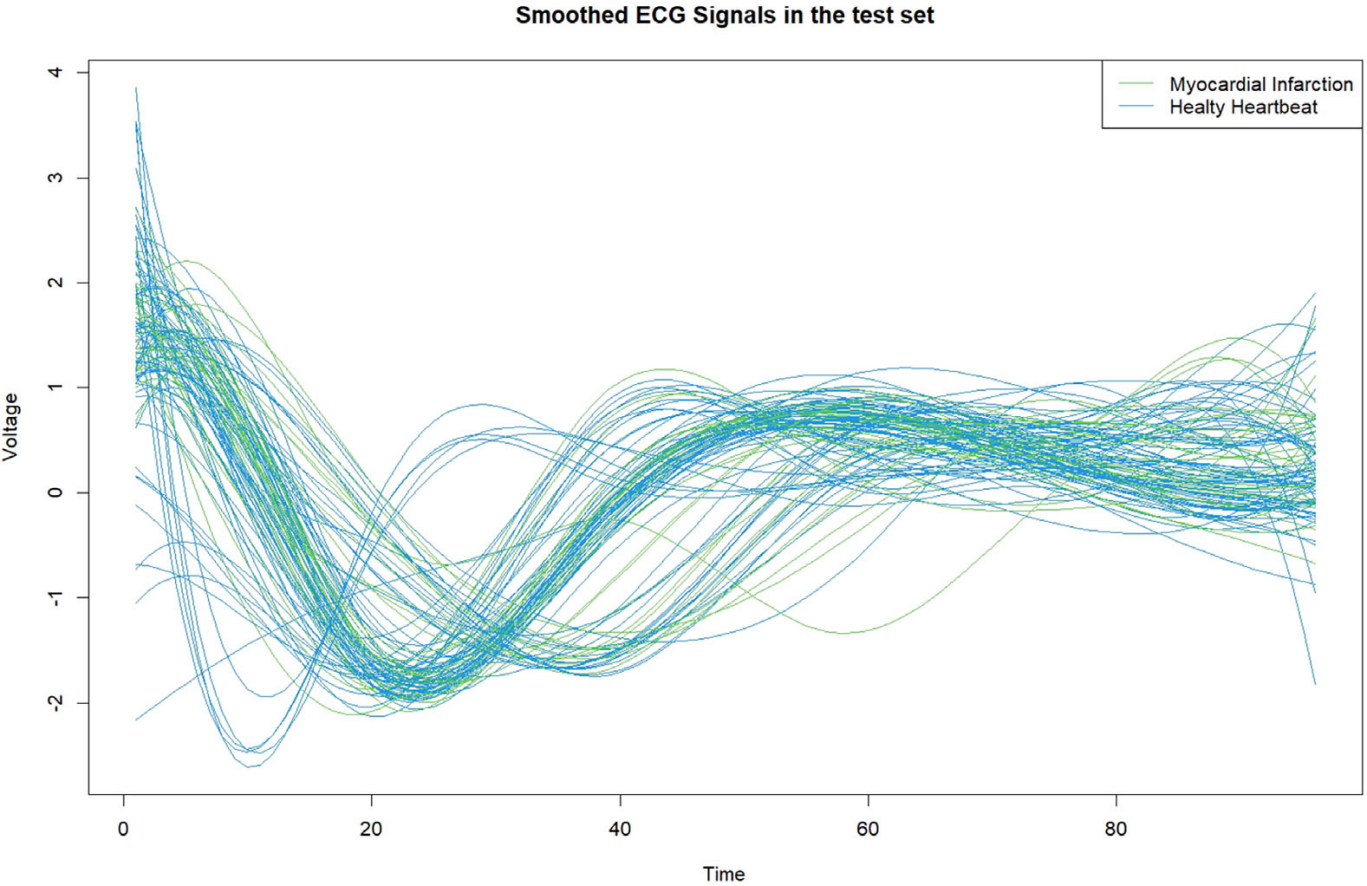
Smoothed ECG curves in the test dataset

Figures 1 and 2 show the smoothed versions of the original signals in the training and test sets computed using Equation (4) via the *fda* and *fda.usc* R packages.^29^, ^43^ The blue signals represent healthy patients (NH), whereas the green curves represent those who had a diagnosis of heart disease (MI).

**FIGURE 3:**
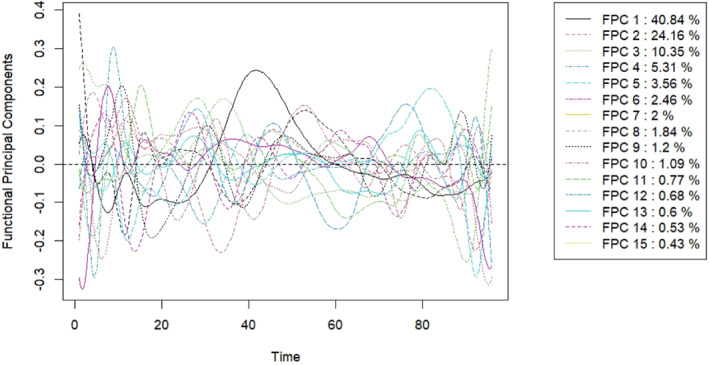
First fifteen FPCs of the original ECG curves of the training set

This first part of the application focuses on the FPCs approach to build FCTs and FRF. Figure 3 considers the first fifteen FPCs. The variability explained by each FPC is shown in the legend. The first three FPCs explain about 75% of the total variability. We remark that, in this framework, the traditional ways of choosing the number of FPCs are not helpful. Indeed, FPCs explaining little variability are often decisive in discriminating the outcome classes and thus are essential in the construction of the FCT. Effectively, the first FPC, which by construction is the one that catches most of the variability (see the black curve in the central part of the time domain), is rarely crucial in FCT. As Figure 3 remarks, each FPC explains different parts of the time domain differently and can be helpful to discriminate the classes of the outcome in the context of FCTs.

**FIGURE 4:**
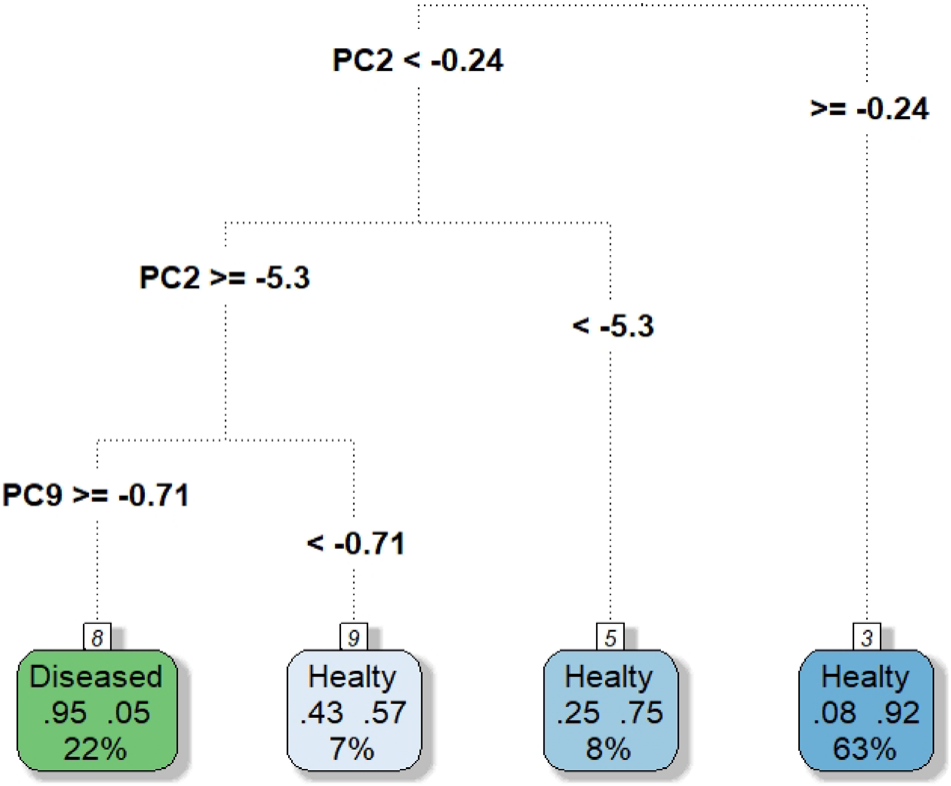
Pruned FCT^44^ based on the FPCs of the ECG200 dataset. The green leaves indicate a classification as diseased, while the blue leaves lead to the classification of the subjects as healthy. The relative frequencies of subjects of the two groups that make up the terminal leaf are indicated in the boxes. The percentage indicated at the bottom of the box indicates the fraction of people classified following the decision rule that leads to that terminal leaf

A non‐pruned tree (available as supplementary material) is rarely useful for practical purposes; in fact, when the FCT incurs in overfitting, it will have poor performance in classifying different datasets. For this reason, the pruning phase,^39^ based on the search for an optimal trade‐off between complexity and accuracy, is fundamental. The cost‐complexity pruning via cross‐validation is performed using the R package *rpart*.^35^ Figure 4 shows the pruned FCT built using the FPCs' scores as features via the *rpart.plot* R package.^44^ The cut on a specific value of a FPCs' score determines the split of a node. Among all the possible FPCs and splitting score values, the one that maximizes the decrease of impurity of the node is chosen. The second FPC is the most important feature in our functional classifier. As expected, the first FPC is not essential for discriminating the classes, as it captures a variability common to many curves of different classes. Curiously, the ninth FPC appears to be important in improving the separation rule. This last result proves that, in this context, the most discriminant FPCs are not necessarily the ones explaining the highest inertia according to the classical criterion of PCs selection.

**FIGURE 5:**
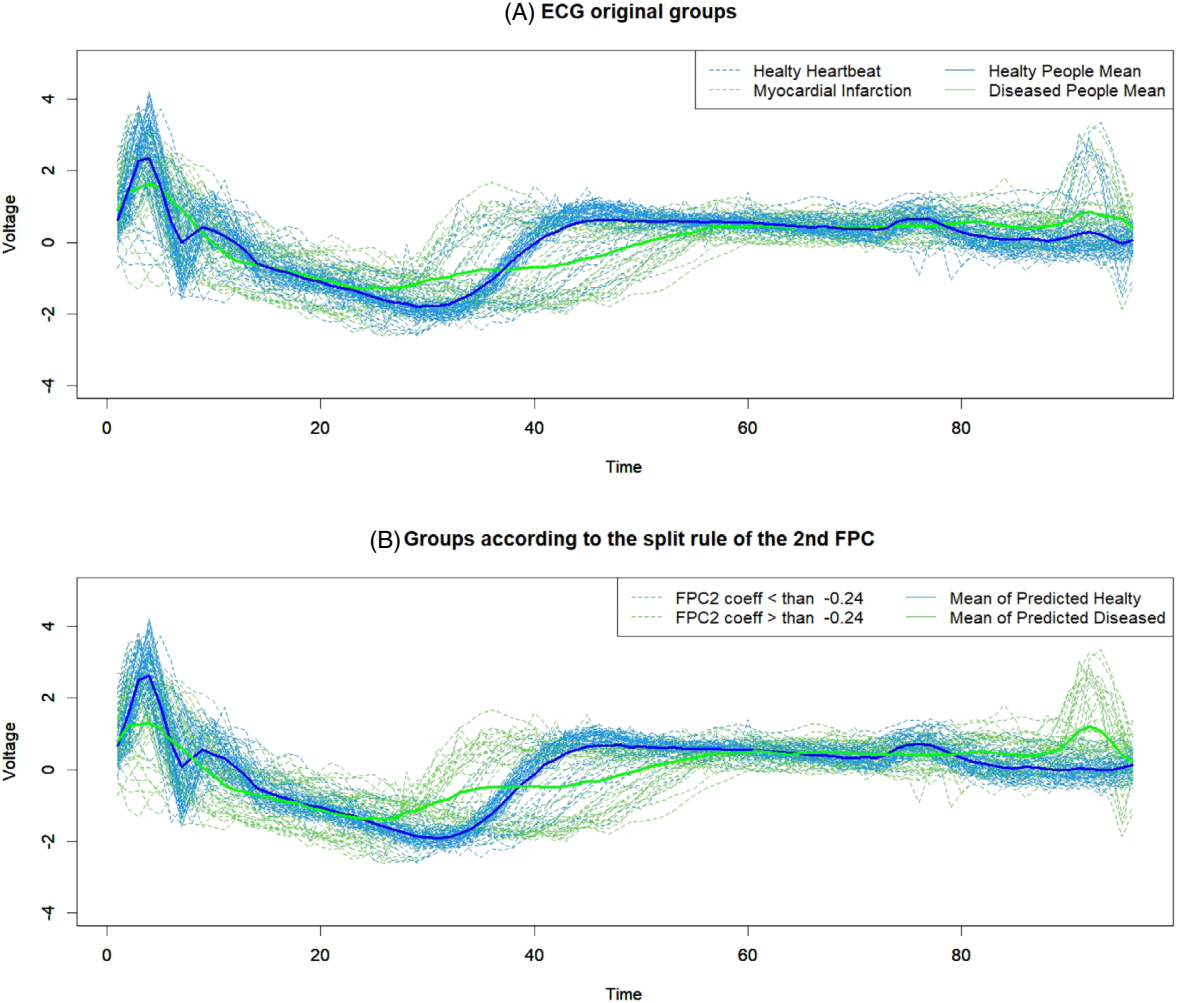
The original ECG groups and the predicted ECG groups according to the first split rule of the root node

Figure 5 explains in detail the first separation rule (root node) dictated by the second FPC. Figure 5A shows the two original groups and their functional means, while Figure 5B depicts the predicted groups applying only the first separation rule and their functional means. Evidently, the second FPC has great power in distinguishing between NH and MI because the two figures appear to be similar. However, beyond the first evidence, there are many curves that are still poorly classified.

**FIGURE 6:**
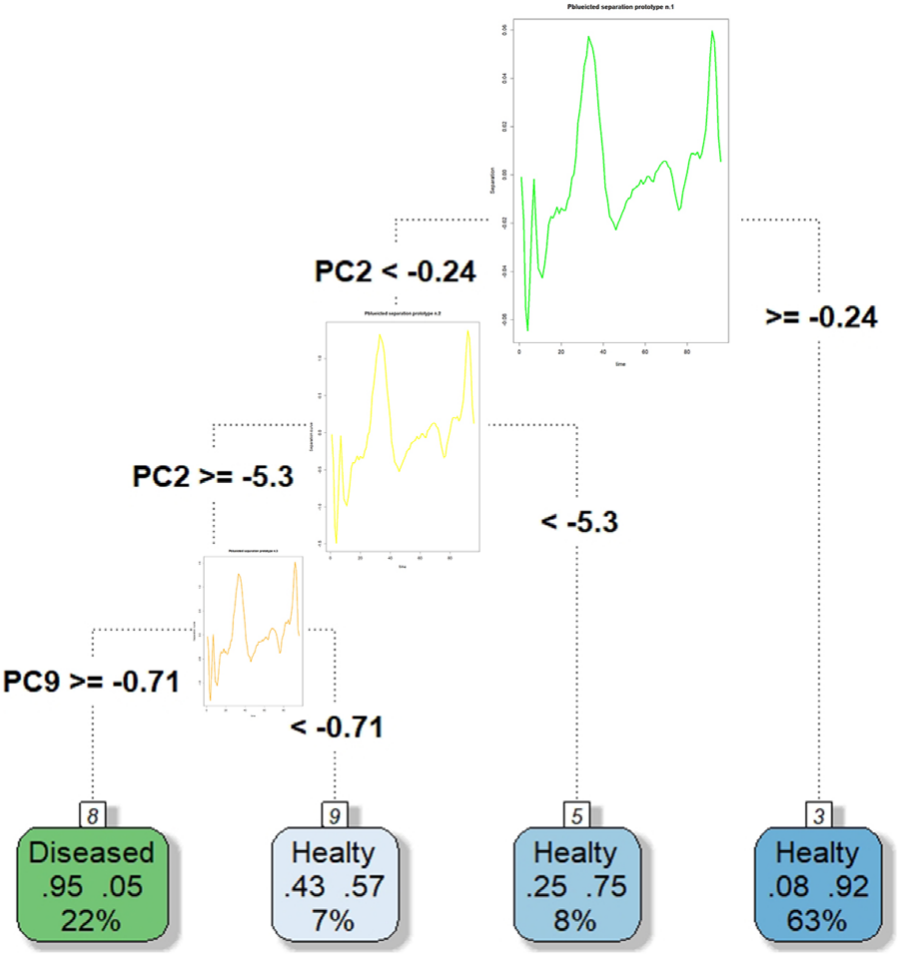
Theoretical splitting curves (TSCs) of the FCT obtained using the three splits based on FPCs

**FIGURE 7:**
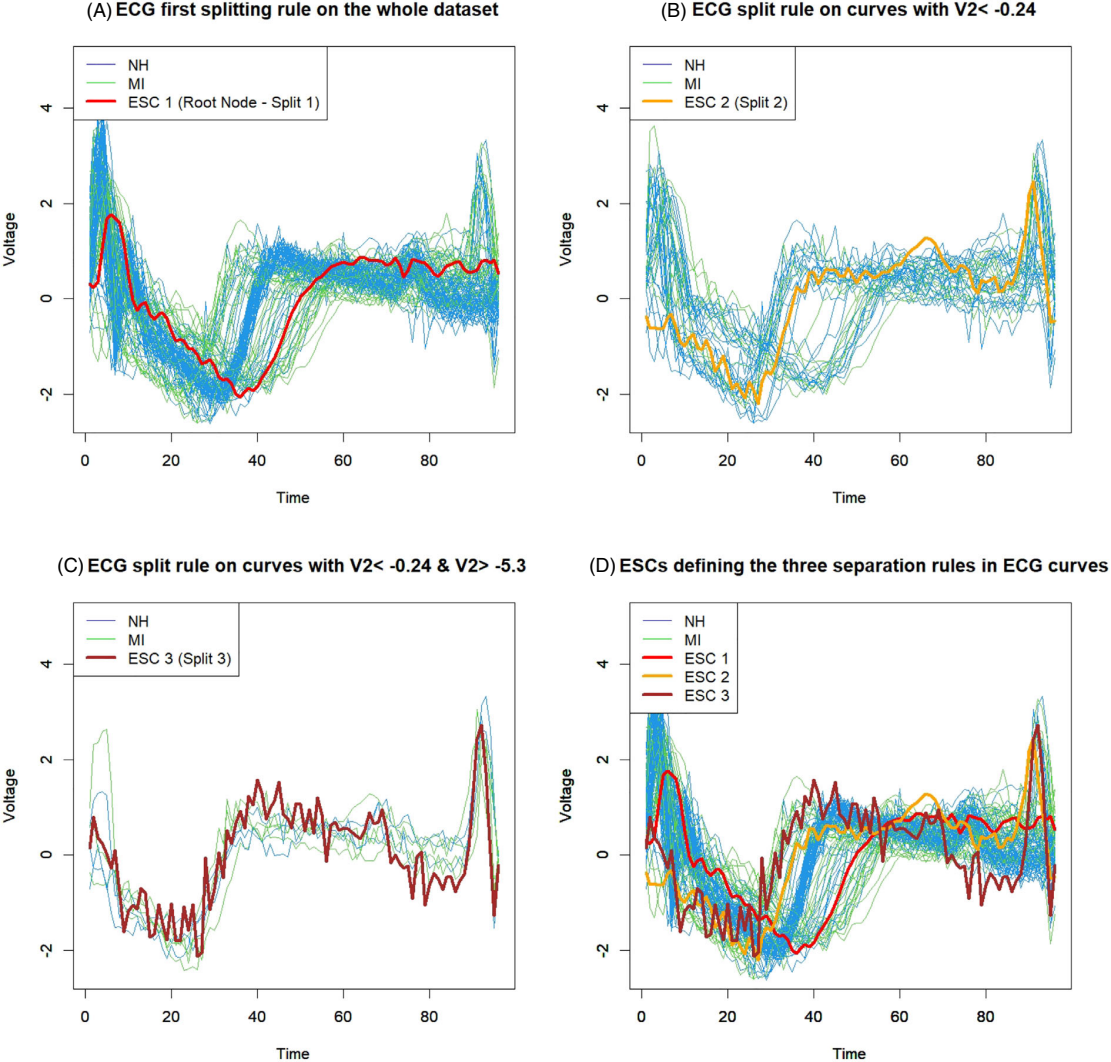
Empirical splitting curves (ESCs) of the FCT obtained using the three splits based on FPCs

Figure 6 illustrates the TSCs obtained as a functional linear combination using the three splitting score values. The three TSCs computed according to Equation (19) are inserted in the representation of the FCT to support the understanding of the classification strategy. Although the three TSCs seem to have high variability over time, this effect is apparent and only due to the increased scale of the ordinate axis. In reality, these curves are very flat. Therefore, when plotted together with the original curves, TSCs do not help to explain the separation rule in terms of curves existing in the data. For this reason, we introduce the ESCs showed in Figure 7. Figure 7A–C exhibit the three ESCs based on the three different classification rules recommended by the FCT. The red curve indicates the ESC derived from the root node split based on the threshold $\nu_{02}=-0.24$. The orange function is the ESC based on the split of the subset of curves satisfying the condition $\nu_{02}<-0.24$. Using the latter subset, the orange curve discriminates those functions based on the threshold value $\nu_{02}=-5.3$. The brown curve, in Figure 7C, describes the third ESC applied to the subset of curves satisfying the conditions $\nu_{02}<-0.24$ and $\nu_{02}\geq -5.3$. On the latter set of curves, the splitting rule based on the split value $\nu_{09}=-0.71$ is applied. Figure 7D illustrates the three ESCs overlapping the whole dataset to help to interpret the separation rules.

**FIGURE 8:**
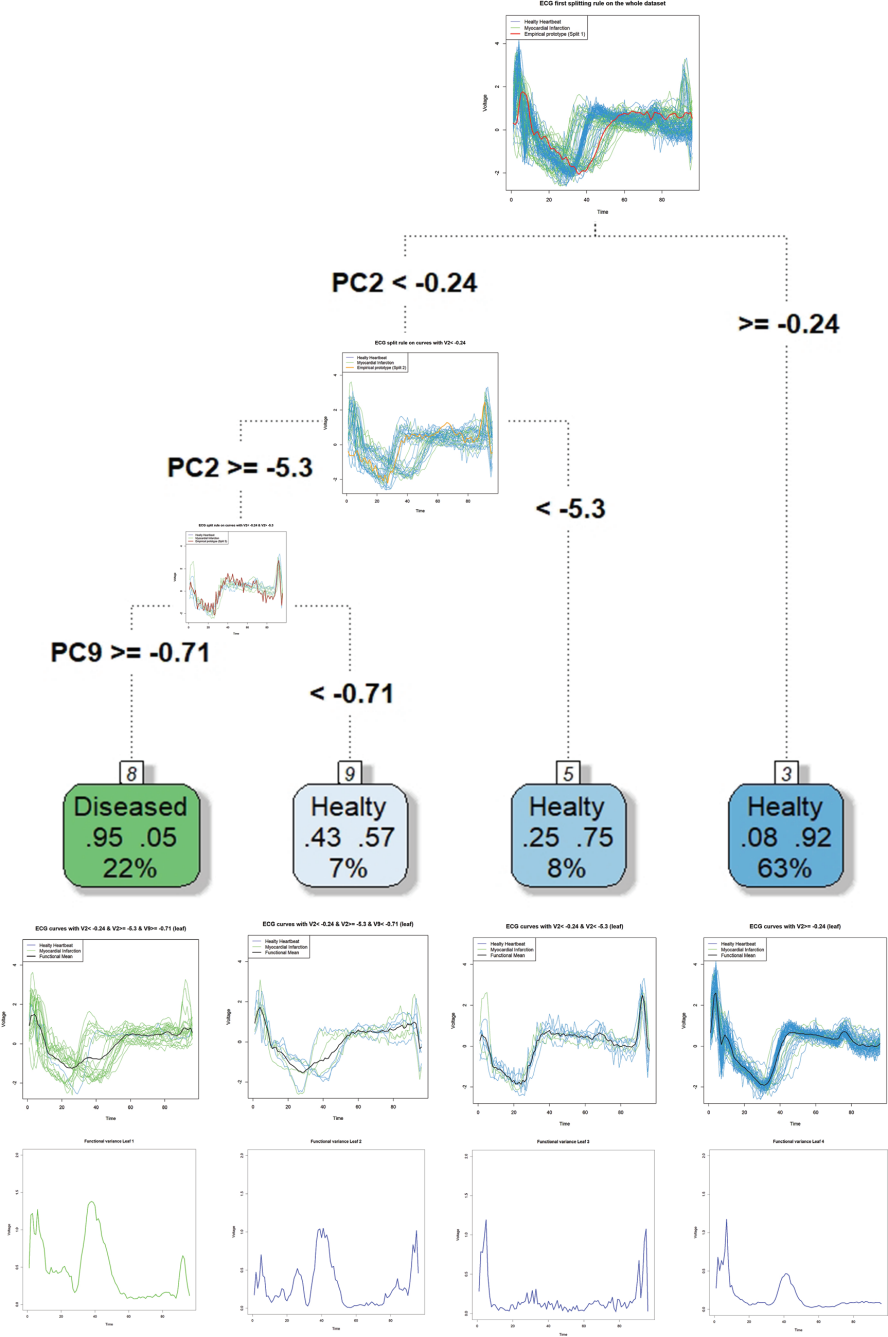
Details of the splitting rules generated by the FCT in terms of ESCs and leaves deviance. The functional deviance of the leaf is plotted below each terminal node. The blue and green colors are linked to the final predicted class of each terminal node. Green = MI; Blue = NH

Figure 8 summarizes all the aspects of the splitting rules produced by the FPC in terms of ESCs. The graph also shows the structure of each terminal node displaying the curves, the functional mean, original classes, and functional deviance of each leaf. The read of Figure 8 highlights interesting results. Indeed, the first leaf (reading from left to right) is mainly composed of curves representing people with a cardiac problem, and thus the overall predicted class is “*Disease*.” Effectively, 95% of the functions in that leaf benefits from an accurate prediction of MI. In other words, the first leaf is characterized by 95% of TPs and 5% of FPs. Figure 8 also shows the functional deviance of the leaves, computed using Equation (21). In addition to the classical measures to estimate the impurity of the terminal nodes, the functional deviance of the leaves can be used to evaluate the internal variability of the single leaf over the whole domain. The functional deviance of the first leaf highlights an interesting peculiarity of this subset of curves. Despite the final classification of the curves in this leaf is quite precise, there is a large functional variability in the middle part of the time domain. This circumstance suggests two different patterns present within the node and that the MI condition does not have only one type of functional form. The leaves number two and three are composed of seven and eight curves, respectively. The dominant prediction for those curves is “*Healthy*,” but the impurity of those terminal nodes is high because there are high percentages of curves also representing “*Diseased*” people. For this reason, the percentages of FNs in these terminal nodes are 43% and 25%, but, in absolute numbers, the contribution to the increase of the misclassification error rate is quite low. Leaf 4 is dominated by TNs, and thus the overall predicted class is “*Healthy*.” Only 8% of the leaf is composed by FNs, and thus the classification rule dictated by the first split rule based on the second FPC is quite good; in fact, it separates 63% of individuals, inserting them in a terminal node. As for leaf 1, the reading of the functional deviance of leaf 4 is quite interesting. Despite the high purity of this node, we observe an intensified variability at the beginning and in the middle part of the time domain. It could be a clue to the presence of two different patterns of healthy people that experienced cardiologists should carefully interpret to understand the meaning of these differences over the time domain. As for leaf 1, these differences could also be due to patient characteristics such as age, sex, comorbidities, or other possible covariates. Therefore, medical advice is essential to interpret this high variability in quite pure leaves.

**FIGURE 9:**
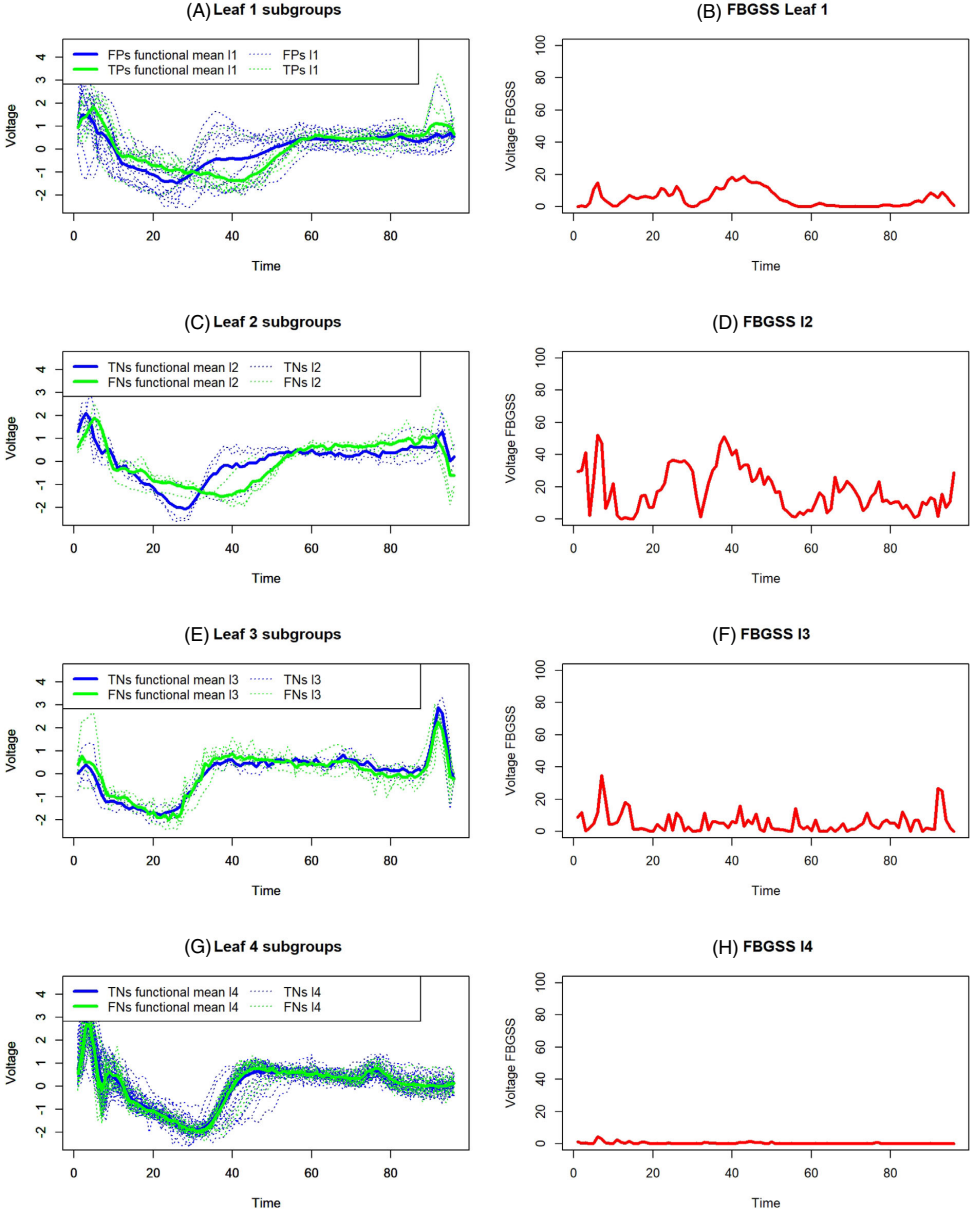
Details of leaves composition: Functional group means and relative FBGSS between TP‐FP or TN‐FN for the four leaves (FBGSS = functional between groups sum of squares)

Figure 9 provides details of the relative FBGSS of the four leaves. Particularly, Figure 9A,B provides details of leaf 1. Because the final prediction of leaf 1 is “*Disease*,” this terminal node is composed of two types of curves, that is, TPs and FPs. TPs in leaf 1 are those curves describing ECGs of people that are really diseased and thus are correctly classified. FPs are those individuals whose curves should be classified as “*Healthy*” but they are not; thus, the latter functions are in the terminal node 1 for a mistake. Now, the most critical question is the following. Which are the similarities and dissimilarities between the functions of these two subgroups? In other words, which are the similarities that led to putting those people into the same terminal node? Furthermore, which are the dissimilarities that have been neglected, leading to a misclassification error? Figure 9A highlights the functional means of FPs and TPs in leaf 1. Instead, Figure 9B presents the plot of the relative FBGSS between FPs and TPs computed using Equation (25). According to the chart, we can state that the classification rule that led to assign these curves in the same terminal node neglected that these functions are slightly diverse in the first half of the time domain. Evidently, the similarity between FPs and TPs curves in the second part of the time domain led to uncertainty in identifying the labels of the curves. Thus, the pruning of the FCT pushed to neglect some features able to discriminate some curves in the first half of the time domain. Figure 9C–F depict the subgroups of TNs and FNs, their functional means, and the relative FBGSSs, respectively. Figure 9G,H show the details of leaf 4. The last terminal node directly comes from the split of the root node (see Figure 8). 92% of the curves in leaf 4 are TNs and describe “*Healthy*” patients that are correctly classified. However, 8% of the curves in leaf 4 are FNs. The plot of the relative FBGSS in Figure 9H helps us to explain why FNs are in the same terminal node of TNs. Effectively, the plot of FBGSS stresses that the groups are very similar, and it is very hard to discriminate between them. The only minor difference between TNs and FNs in leaf 4 can be found in the first quarter of the time domain, but their divergence is too small to be detected.

**FIGURE 10:**
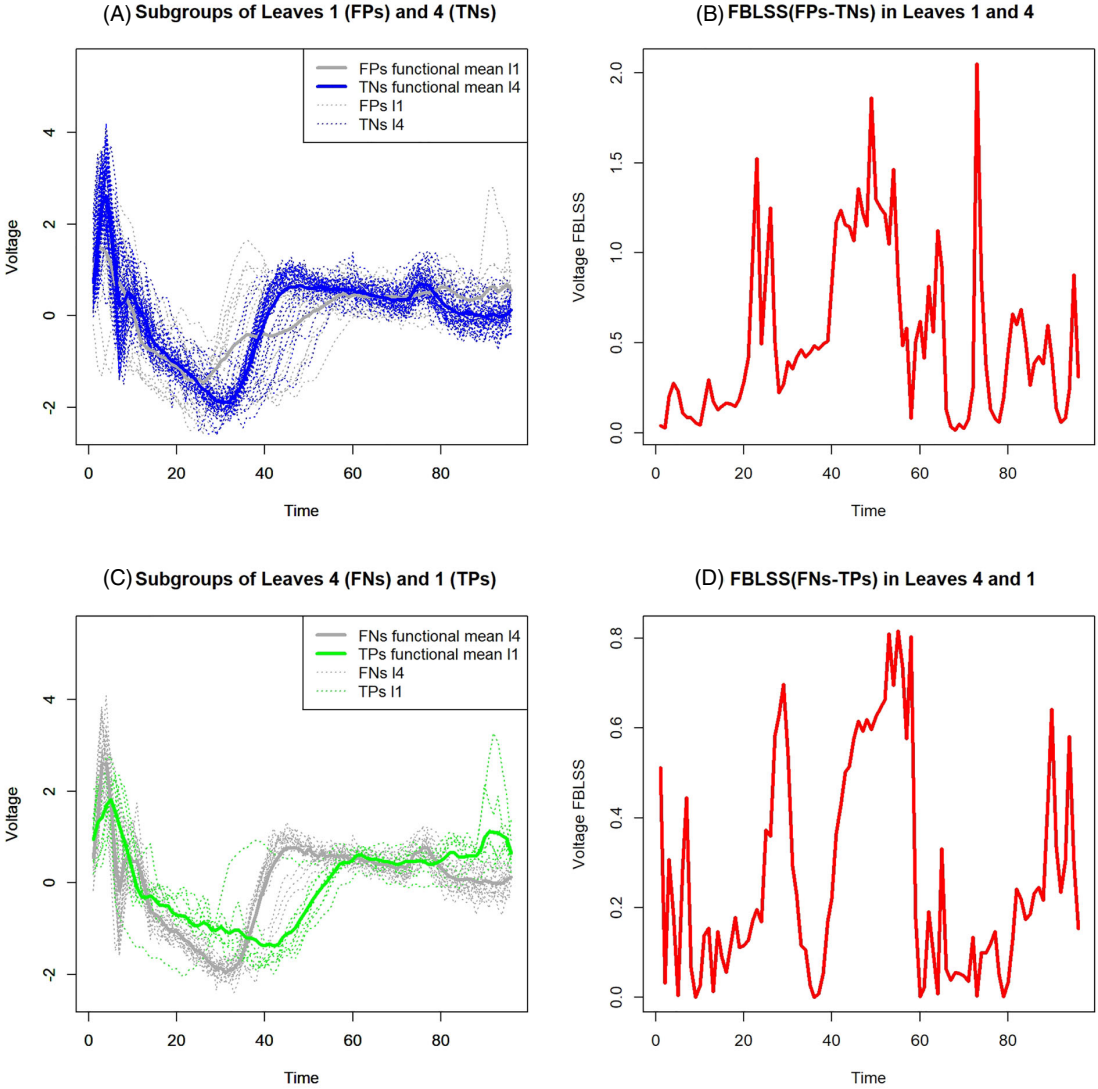
FBLSS 1 vs 4, and vice versa, that is, FPs vs TNs, and FNs vs TPs (FBGLS = functional between leaves sum of squares)

Figure 10 provides a detailed view of the cross‐comparison between leaves 1 and 4. The latter leaves contain the highest number of curves; indeed, the leaf 1 considers 22% and leaf 4 contains 63% of the total number of curves. Moreover, they can both used as reference leaves because they are also the most accurate in predicting the “*Disease*” and “*Healthy*” classes, respectively (see Figure 8). Therefore, we omit discussing all the possible cross‐comparisons of terminal nodes, and we focus only on leaves 1 and 4. Specifically, Figure 10A,B presents FPs of leaf 1 and TNs of leaf 4 functional means, and their relative FBLSS. Figure 10C,D shows FNs of leaf 4 and TPs of leaf 1 functional means, and their relative FBLSS. The latter charts allow us to try answering the most challenging issues. The first problem (Figure 10A,B) is to capture the difference between FPs in leaf 1 and TNs in leaf 4. In other words, we aim to explain the functional variability between healthy people that are correctly predicted and healthy people that are not correctly predicted by considering the most dominant decision rules in terms of the number of individuals in the leaves with opposite class predictions. The charts stress that the bad predictions of FPs in leaf 1 is probably due to a peculiar shapes of these curves in three parts of the domain (red curve's peaks). Indeed, the gray curve (FPs' functional mean in leaf 1) is very different from the blue function (TNs' functional mean in leaf 4), especially in the central area of the domain, which appears to be decisive for getting why FPs are badly assigned to leaf 1 instead of leaf 4. The second issue is the opposite, that is, trying to explain why some diseased patients (FNs in leaf 4) are assigned to a leaf that is mainly composed by healthy people and which is their functional between variability with respect to diseased people that are correctly classified in leaf 1 (TPs in leaf 1). Effectively, leaf 1 mainly predicts the disease status with 95% accuracy and thus understanding the difference with respect to this subgroup of TPs can help to detect peculiarities/anomalies in some ill patients, and probably also different patterns of disease. It is evident that, from a functional point of view, the sick and poorly classified patients of leaf 4 are very different from the well classified sick patients of leaf 1. The biggest difference is evidenced by the FBLSS peak exactly in the center of the time domain, where we observe a constant high value of FBLSS. Effectively, also looking at the green curve (the prototype of TPs in leaf 1) in Figure 10C, it appears to be much lower than the gray curve (FNs in leaf 4) in the central part of the domain. It would seem that two different patterns characterize MI patients, and one of these patterns is difficult to recognize because it resembles the pattern of healthy people (the gray curve).

Because FRF provides an improvement over FBG, we present only the results of the FRF. Indeed, in the application of FBG with the ECG200 dataset, the FCTs are all dominated by the second FPC and, therefore, very correlated. As discussed in Section 4, in FBG, FCTs are always very similar because at the top of the FCT there are always the same more discriminating features. Thus, we focus on FRF, which also produces the best results in accuracy and variance reduction. Using the FRF algorithm, the FCTs are not all governed by the second FPC. Indeed, at the top of the FCTs in the forest, there are often different FPCs (a picture of the first nine FCTs obtained is attached as supplementary material).

**FIGURE 11:**
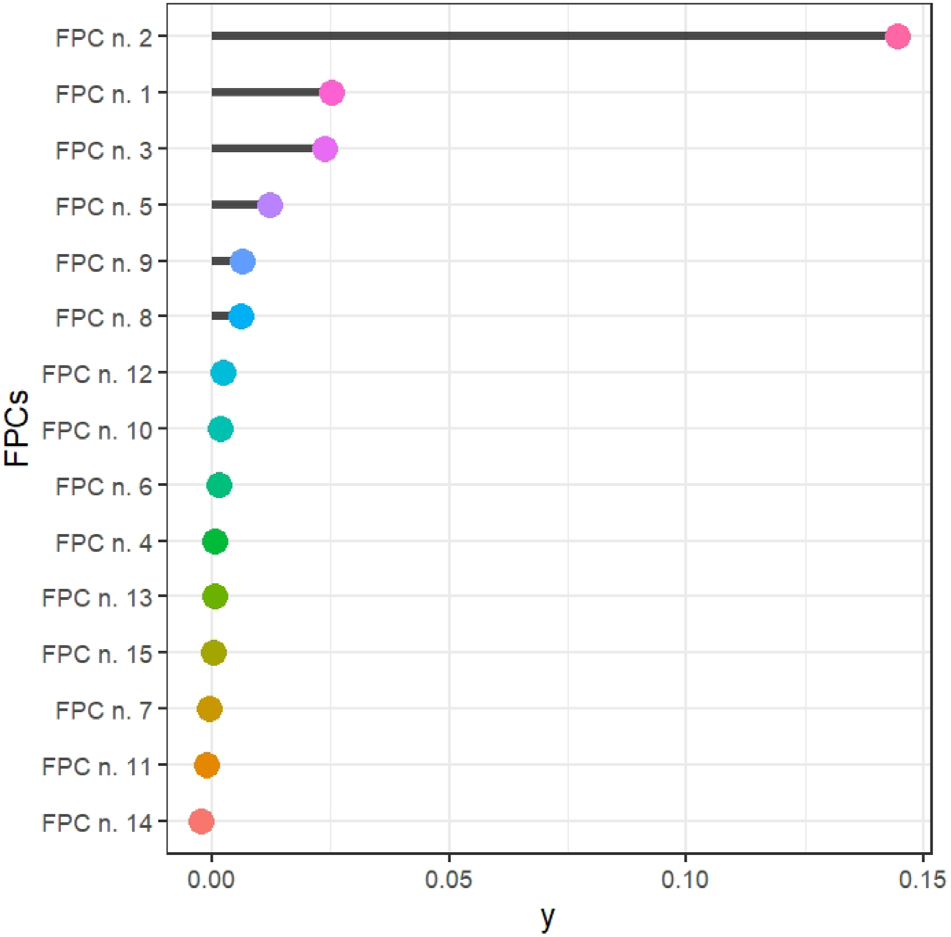
Interpreting the importance of FPCs for the FRF classifier based on FPCs (mean decrease accuracy index)

Figure 11 illustrates the influence of the FPCs in the FRF classifier based on the mean decrease accuracy index.^33^, ^35^ Unquestionably, the second FPC is the most powerful in distinguishing the classes of the outcome; nevertheless, the FPCs n. 1, 3, 5, and 9 also play an influential role in enhancing the accuracy of the functional classifier.

We implemented the FRF algorithm for different sizes of the forest and different numbers of FPCs by testing the accuracy both applying the OOBFD misclassification error estimation and using the test set (see Tables 1 and 2). A comparison with other competitive functional classification techniques is presented in Tables 3, 4, 5. The best performance computed using the OOBFD strategy on the training set is 89.00% with 12 FPCs and 9 trees. Instead, Table 2 exhibits the accuracy of the FRF classifier using the test set. The best accuracy on the test set is 93.00% with 9 FPCs and 9 FCTs. The world top accuracy reached for this dataset was 89.05% with the BOSS algorithm.^42^ Hence, the FRF classifier based on FPCs beats the previous world record set by the BOSS algorithm.

**TABLE 1:** Accuracy for the ECG200 dataset using the training set: Accuracy of the FRF classifier according to different sizes of the forest and different numbers of FPCs

	Number of functional principal components involved in the classifier FRF													
No. of trees	2	3	4	5	6	7	8	9	10	11	12	13	14	15
2	77.78	78.57	90.16	77.05	77.78	80.65	85.96	76.92	72.41	80.00	80.00	74.24	72.13	82.26
3	76.54	80.28	82.43	73.08	76.39	81.16	83.33	78.26	75.71	76.32	79.17	76.32	77.46	71.83
4	84.71	74.07	72.29	80.49	80.25	78.65	79.52	79.76	76.14	75.95	76.19	75.00	80.25	79.31
5	82.80	73.91	86.81	79.78	80.00	79.35	76.40	77.78	78.13	74.44	74.44	74.74	75.58	80.00
6	77.42	83.33	82.11	75.79	80.21	74.49	78.95	84.44	79.57	74.74	80.85	79.35	70.97	82.11
7	85.42	82.65	84.69	78.79	75.51	84.38	79.79	74.47	75.79	79.38	81.63	79.79	72.34	83.16
8	77.55	83.51	77.78	78.72	75.51	78.72	75.51	78.12	81.63	77.32	79.17	80.41	72.83	72.73
9	82.65	83.33	85.86	82.65	73.20	80.41	78.57	76.00	82.65	75.76	82.65	79.80	79.59	80.41
10	77.55	79.00	79.80	80.61	77.78	81.63	74.49	77.55	80.00	76.53	76.53	81.82	87.76	75.51
11	83.00	77.00	83.00	75.00	81.82	73.00	78.00	82.00	74.00	73.00	79.80	82.00	86.00	78.00
12	78.00	82.83	87.00	81.82	75.00	75.00	77.00	75.51	80.00	80.00	82.65	83.84	86.00	70.71
13	86.00	82.00	81.00	77.78	76.29	85.00	77.00	79.00	83.00	79.00	80.00	84.85	80.00	82.00
14	78.79	78.00	83.00	81.00	77.00	80.00	80.81	81.00	84.00	77.00	84.00	78.00	78.00	84.00
15	82.00	80.00	80.00	80.00	81.00	81.00	82.00	83.00	82.00	76.00	79.00	79.00	80.00	80.00
16	80.00	85.00	81.00	80.00	82.00	84.00	78.79	78.00	80.00	79.00	80.81	82.00	78.00	80.00
17	82.00	87.00	85.00	79.00	79.00	76.77	78.00	77.00	80.00	75.00	81.00	82.00	73.00	87.88
18	84.00	80.00	87.00	76.00	84.00	79.00	81.00	84.00	84.00	86.00	82.00	76.00	77.00	78.00
19	84.00	83.00	78.00	82.00	83.00	80.00	78.00	80.00	84.00	83.00	86.00	83.00	82.00	80.00
20	84.00	84.00	86.00	85.00	83.00	81.00	80.00	78.00	82.00	82.00	81.00	83.00	77.00	84.00
21	81.00	86.00	80.00	86.00	79.00	82.00	80.00	78.00	80.00	74.00	88.00	82.00	81.00	83.00
22	83.00	85.00	83.00	82.00	82.00	76.00	80.00	77.00	83.00	82.00	83.00	80.00	82.00	81.00
23	84.00	85.00	83.00	80.00	85.00	84.00	81.00	78.00	84.00	81.00	81.00	82.00	77.00	79.00
24	85.00	85.00	83.00	84.00	84.00	83.00	86.00	84.00	88.00	86.00	83.00	77.00	78.00	83.00
25	79.00	89.00	85.00	81.00	85.00	78.00	82.00	83.00	84.00	81.00	79.00	80.00	83.00	80.00
26	81.00	83.00	85.00	84.00	79.00	83.00	81.00	84.00	84.00	85.00	83.00	83.00	79.00	81.00
27	84.00	82.00	83.00	82.00	84.00	81.00	82.00	83.00	83.00	80.00	79.00	80.00	85.00	84.00
28	87.00	84.00	84.00	85.00	82.00	83.00	84.00	82.00	79.00	82.00	83.00	83.00	82.00	80.00
29	83.00	86.00	86.00	82.00	82.00	84.00	84.00	83.00	81.00	79.00	83.00	80.00	80.00	84.00
30	88.00	84.00	83.00	81.00	87.00	85.00	79.00	82.00	84.00	82.00	79.00	83.00	82.00	80.00
31	84.00	86.00	85.00	85.00	84.00	84.00	76.00	81.00	80.00	83.00	77.00	80.00	83.00	84.00
32	82.00	79.00	85.00	83.00	79.00	82.00	82.00	84.00	84.00	83.00	87.00	87.00	82.00	83.00
33	84.00	80.00	79.00	85.00	83.00	84.00	85.00	80.00	81.00	84.00	86.00	84.00	81.00	85.00
34	84.00	84.00	81.00	85.00	84.00	83.00	79.00	81.00	82.00	80.00	80.00	81.00	81.00	81.00
35	84.00	82.00	85.00	82.00	84.00	81.00	84.00	82.00	84.00	82.00	80.00	81.00	88.00	83.00
36	84.00	84.00	83.00	85.00	86.00	82.00	80.00	81.00	83.00	85.00	82.00	83.00	83.00	87.00
37	80.00	83.00	83.00	78.00	84.00	81.00	84.00	82.00	83.00	83.00	82.00	84.00	83.00	78.00
38	83.00	83.00	85.00	85.00	86.00	85.00	79.00	83.00	85.00	85.00	82.00	84.00	80.00	85.00
39	87.00	83.00	84.00	85.00	84.00	84.00	78.00	83.00	81.00	82.00	85.00	83.00	81.00	84.00
40	85.00	86.00	83.00	86.00	83.00	80.00	80.00	83.00	82.00	82.00	83.00	80.00	85.00	83.00
45	86.00	82.00	83.00	82.00	83.00	82.00	81.00	81.00	85.00	83.00	81.00	81.00	83.00	81.00
50	84.00	80.00	81.00	84.00	79.00	81.00	81.00	79.00	81.00	82.00	79.00	82.00	83.00	83.00

*Note*: The best accuracy on the training set is 89.00% with 12 FPCs and 9 trees.

**TABLE 2:** Accuracy for the ECG200 dataset using the test set: Accuracy of the FRF classifier according to different sizes of the forest and different numbers of FPCs

	Number of functional principal components involved in the classifier FRF													
No. of trees	2	3	4	5	6	7	8	9	10	11	12	13	14	15
2	74.00	73.00	83.00	79.00	68.00	85.00	68.00	74.00	75.00	74.00	72.00	75.00	74.00	74.00
3	78.00	76.00	81.00	84.00	82.00	82.00	74.00	82.00	82.00	71.00	78.00	78.00	73.00	73.00
4	78.00	79.00	78.00	72.00	81.00	79.00	83.00	84.00	83.00	81.00	86.00	76.00	78.00	84.00
5	74.00	75.00	84.00	82.00	83.00	87.00	81.00	81.00	82.00	82.00	86.00	83.00	82.00	75.00
6	78.00	82.00	70.00	84.00	84.00	79.00	82.00	84.00	81.00	79.00	86.00	84.00	85.00	84.00
7	74.00	80.00	82.00	82.00	86.00	83.00	82.00	84.00	83.00	82.00	85.00	82.00	82.00	82.00
8	79.00	81.00	85.00	79.00	84.00	78.00	84.00	89.00	84.00	86.00	85.00	90.00	78.00	87.00
9	73.00	82.00	83.00	85.00	84.00	78.00	83.00	93.00	85.00	89.00	86.00	82.00	88.00	82.00
10	77.00	82.00	80.00	84.00	84.00	79.00	81.00	83.00	85.00	87.00	88.00	85.00	87.00	80.00
11	80.00	75.00	84.00	85.00	85.00	83.00	86.00	85.00	88.00	82.00	84.00	84.00	81.00	81.00
12	79.00	82.00	80.00	84.00	83.00	79.00	82.00	80.00	84.00	92.00	82.00	86.00	81.00	82.00
13	79.00	81.00	84.00	88.00	77.00	83.00	83.00	83.00	86.00	87.00	87.00	84.00	82.00	85.00
14	78.00	85.00	83.00	81.00	85.00	80.00	83.00	87.00	85.00	85.00	86.00	81.00	89.00	81.00
15	79.00	80.00	83.00	87.00	85.00	82.00	84.00	86.00	84.00	85.00	81.00	85.00	88.00	82.00
16	78.00	81.00	82.00	84.00	85.00	81.00	84.00	87.00	87.00	88.00	90.00	85.00	85.00	83.00
17	80.00	82.00	85.00	82.00	86.00	83.00	81.00	84.00	86.00	88.00	83.00	85.00	82.00	86.00
18	79.00	80.00	81.00	85.00	84.00	85.00	87.00	86.00	89.00	91.00	87.00	88.00	87.00	82.00
19	81.00	78.00	80.00	82.00	84.00	82.00	84.00	84.00	86.00	86.00	82.00	83.00	87.00	88.00
20	79.00	78.00	79.00	84.00	84.00	81.00	82.00	87.00	85.00	89.00	88.00	81.00	86.00	84.00
21	77.00	78.00	79.00	86.00	82.00	84.00	86.00	88.00	85.00	84.00	89.00	89.00	83.00	81.00
22	81.00	79.00	84.00	81.00	83.00	84.00	79.00	84.00	87.00	88.00	89.00	82.00	85.00	87.00
23	80.00	74.00	84.00	81.00	86.00	83.00	88.00	83.00	85.00	85.00	89.00	83.00	85.00	88.00
24	76.00	80.00	82.00	82.00	81.00	85.00	88.00	87.00	86.00	85.00	90.00	85.00	85.00	85.00
25	78.00	79.00	85.00	85.00	85.00	83.00	84.00	85.00	87.00	87.00	87.00	82.00	86.00	86.00
26	79.00	77.00	82.00	86.00	80.00	82.00	82.00	87.00	88.00	85.00	86.00	88.00	86.00	90.00
27	80.00	76.00	80.00	85.00	81.00	84.00	84.00	87.00	83.00	89.00	82.00	86.00	88.00	85.00
28	79.00	77.00	79.00	82.00	85.00	84.00	84.00	85.00	86.00	89.00	86.00	87.00	87.00	89.00
29	79.00	81.00	79.00	86.00	82.00	84.00	84.00	87.00	87.00	89.00	84.00	82.00	89.00	86.00
30	78.00	81.00	83.00	84.00	82.00	86.00	86.00	86.00	89.00	88.00	84.00	88.00	87.00	84.00
31	78.00	81.00	80.00	86.00	85.00	82.00	89.00	88.00	86.00	85.00	85.00	89.00	88.00	87.00
32	77.00	77.00	85.00	84.00	82.00	82.00	85.00	86.00	88.00	93.00	87.00	90.00	87.00	89.00
33	78.00	80.00	80.00	84.00	85.00	83.00	81.00	83.00	88.00	86.00	88.00	83.00	84.00	90.00
34	80.00	84.00	83.00	81.00	84.00	84.00	85.00	86.00	85.00	87.00	83.00	84.00	86.00	87.00
35	79.00	79.00	82.00	84.00	83.00	86.00	82.00	87.00	83.00	88.00	86.00	87.00	87.00	85.00
36	80.00	81.00	82.00	82.00	85.00	83.00	83.00	87.00	83.00	87.00	89.00	88.00	88.00	88.00
37	79.00	80.00	83.00	87.00	84.00	83.00	83.00	85.00	88.00	90.00	88.00	84.00	84.00	84.00
38	77.00	81.00	81.00	83.00	83.00	84.00	86.00	84.00	88.00	87.00	88.00	86.00	88.00	85.00
39	78.00	80.00	80.00	83.00	84.00	82.00	86.00	88.00	87.00	88.00	88.00	86.00	85.00	87.00
40	79.00	80.00	81.00	81.00	85.00	84.00	85.00	88.00	84.00	89.00	89.00	87.00	86.00	85.00
45	79.00	83.00	81.00	83.00	85.00	86.00	84.00	86.00	90.00	89.00	86.00	87.00	86.00	86.00
50	78.00	79.00	83.00	85.00	86.00	85.00	83.00	86.00	87.00	87.00	87.00	86.00	85.00	86.00

*Note*: The best accuracy on the test set is 93.00% with 9 FPCs and 9 trees, or 11 FPCs and 32 trees. FRF classifier performs better than the other methods. The world top accuracy reached for this dataset was 89.05% with the BOSS algorithm. https://www.timeseriesclassification.com/description.php?Dataset=ECG200

**TABLE 3:** Accuracy for the ECG200: Accuracy of the FRF‐B‐spline classifier according to different sizes of the forest and a fixed number of B‐splines

No. of trees	Accuracy on training set	Accuracy on test set
2	83.33	86.00
3	81.82	81.00
4	80.25	81.00
5	77.42	80.00
6	81.05	86.00
7	82.47	83.00
8	82.65	76.00
9	78.57	81.00
10	80.00	86.00
11	77.78	82.00
12	83.00	81.00
13	85.00	83.00
14	80.00	86.00
15	81.00	81.00
16	84.00	84.00
17	82.00	83.00
18	81.00	80.00
19	81.00	79.00
20	82.00	82.00
21	86.00	83.00
22	87.00	81.00
23	80.00	81.00
24	87.00	83.00
25	87.00	83.00
26	83.00	83.00
27	85.00	80.00
28	83.00	86.00
29	83.00	83.00
30	83.00	82.00
31	84.00	79.00
32	84.00	81.00
33	84.00	83.00
34	82.00	81.00
35	81.00	82.00
36	83.00	83.00
37	85.00	82.00
38	85.00	83.00
39	85.00	81.00
40	84.00	83.00
45	84.00	83.00
50	85.00	82.00

*Note*: The best accuracy on the training set is 87.00% with 29 trees. The best accuracy on the test set is 87.00% with 8 trees.

**TABLE 4:** Accuracy for the ECG200 dataset: Functional classification using the K‐NN classifier of the R package fda.usc

No. of NNs in functional KNN	Accuracy on training set	Accuracy on test set
1	86.00	89.00
3	92.00	91.00
5	88.00	91.00
7	86.00	88.00
9	81.00	86.00
11	78.00	88.00
13	77.00	85.00
15	76.00	82.00
17	75.00	82.00
19	71.00	80.00
21	72.00	76.00
23	74.00	75.00
25	75.00	76.00

*Note*: The best accuracy on the training set is 92.00% with 3 nearest neighbors. The best accuracy on the test set is 91.00% with 3 or 5 nearest neighbors.

**TABLE 5:** Accuracy for the ECG200: Functional classification using depth classifiers of the R package fda.usc

Functional depth measure	Accuracy on the training set	Accuracy on the test set
	Train	Test
RP	78.00	81.00
mode	80.00	79.00
RT	44.00	43.00
FM	76.00	74.00
RPD	83.00	80.00

*Note*: The best accuracy on the training set is 83% with the depth measure “RPD.” The best accuracy on the test set is 81% with the depth measure “RP.” depth.RP computes the random projection depth (see Cuevas et al^17^). depth.mode implements the modal depth (see Cuevas et al^17^). depth.RT implements the random tukey depth (see Cuesta‐Albertos and Nieto‐Reyes^52^). depth.FM computes the integration of an univariate depth along the axis *x* (see Fraiman and Muniz^53^). It is also known as integrated depth. depth.RPD implements a depth measure based on random projections possibly using several derivatives (see Cuevas et al^17^).

To compare the FRF based on FPCs to the FRF based on b‐splines and the most recent and widespread methods to classify functional data, we provide the results of different approaches implemented in the fda.usc R package.^29^ Tables 3, 4, 5 highlight that the other methods are not able to reach the performance of the FRF classifier. Indeed, the best accuracy of the FRF based on b‐splines gives an accuracy of 87.00% on the training set using 29 FCTs, and the same result is achieved using the test set with 8 FCTs. Moreover, Table 4 shows that, using the functional K‐NN classifier of the R package fda.usc, the best outcome on the training set is 92.00% with 3 nearest neighbors whereas the best result on the test set is 91.00% with 3 or 5 nearest neighbors. Finally, using the functional depth classifiers (Table 5), the best accuracy on the training set is 83.00% with the depth measure “*RPD*,” and the best accuracy on the test set is 81.00% with the depth measure “*RP*.” In summary, the FRF classifier based on FPCs shows exciting results and proves to be the best in terms of accuracy with this data when focusing on the test set.

A second application on the ECG200 dataset is performed using a fixed basis system. For brevity, in this case, we just show some essential results. The b‐spline fixed basis system is composed of 98 b‐splines of order 4. The most essential b‐splines in discriminating the classes are the numbers 33, 32, and 34. The accuracy of the b‐splines‐based FRF classifier gives interesting results. The best accuracy on the training set is 87.00% using twenty‐nine trees. The best accuracy on the test set is 87.00% with eight trees. This approach is interesting to reduce the dimensionality and get good accuracy. However, the former is inferior to the data‐driven approach and the BOSS algorithm, which reached the previous record on this dataset. In summary, the interpretation is relatively complicated compared to the data‐driven approach, and the classifier's performance is not up to par. For this reason, we consider the FRF based on FPCs as the “gold standard” of our proposal. Detailed information regarding the pruned FRF using b‐splines are the b‐splines importance in building FRF procedure are available as supplementary material.

### 5.2 Results of the application to the ECG5000 dataset

The ECG5000 dataset^42^ was initially used in the article “*A general framework for never‐ending learning from time series streams*.”^45^ After that, 5000 heartbeats were randomly selected. The training set is composed of 500 patients, and the test set contains 4500 persons. The dataset is made up of five groups of patients with different types of heart disease. The best accuracy on the training set is 97.28% with 4 FPCs and 20 trees. On the other hand, the best accuracy on the test set is 93.64% with 6 FPCs and 40 trees. The world top accuracy reached for this dataset is still 94.61% with the COTE algorithm. Using b‐splines, the best accuracy on the training set is 95.20% with 29 trees, and the best accuracy on the test set is 93.87% with 24 trees. The best accuracy of the functional K‐NN classifier on the training set is 95.00% with nine nearest neighbors. Instead, using the test set is 93.64% with nine nearest neighbors. Regarding the functional depth classifiers of the R package fda.usc, the best result on the training set is 93.6% with the depth measure “mode,” whereas the best accuracy on the test set is 91.87% with the same depth measure. The FRF based on FPCs reaches very high accuracy in this second application by testing the misclassification error rate via the OOBFD strategy. Using the test set, the performances in terms of accuracy are pretty similar if considering FRF with FPCs, FRF with b‐splines, or functional K‐NN. The interpretation described for the ECG200 dataset with two classes can be extended to cases where the number of classes is greater than two. More details about the application on the ECG5000 are available as supplementary materials.

## 6 DISCUSSION AND CONCLUSIONS

Today, thanks to technological progress, we can collect vast amounts of biomedical data. These data often come from medical devices, apps, and different sensors that produce high‐frequency observations, for example, for monitoring the heart with ECGs, controlling brain activities via EEGs, assessing lung function using pulmonary function tests (PFTs), recordings of various types of signals to study sleep disorders. Consequently, recently, many approaches have been developed to deal with this data because traditional statistical methods can fail. One of the most critical issues is, for example, the so‐called curse of dimensionality that is linked with many problematical drawbacks when working with high‐dimensional biological data.

This study offers a classification strategy for high‐dimensional biomedical data that merges FDA and tree‐based procedures. FCTs, functional bagging, and FRF are considered possible extensions of the classical classification trees, bagging, and random forest approach to deal with functional data. The idea of combining FDA and decision trees is relatively novel. Few articles dealt with this topic in recent years, although the literature on supervised classification in the FDA context is very lively. For example, Moller et al^25^ recommended selecting features based on the mean of the function within determined intervals of the domain, whereas ElHaouij et al^46^ and Gregorutti et al^24^ concentrated on the wavelet basis representation with particular applications.

Our approach is quite different and suggests two possible strategies on a data‐driven basis and fixed‐basis systems. However, the objective of the proposed method is multiple. The first goal is connected with the introduction of the FDA because it permits a robust dimensionality reduction with an interpretation linked to different parts of the time domain and quick perception.^2^, ^29^, ^47^ In addition, FDA also provides extra information on the behavior and variability of the curves over time.^7^ Finally, treating functions as single objects permits us to employ some concepts of similarity between statistical units, which are very charming because they only take into account the primary features of the functions.^1^ The second purpose is linked to the introduction of FCTs combined with FDA. In particular, by introducing functional principal components as possible features to train a classification tree, it is possible to apply a dimensionality reduction that offers uncorrelated predictors and reduces unnecessary noise for classification purposes. The third and most innovative element of this research is undoubtedly linked to the functional interpretation of FCTs and their decision rules. By pooling FPCs and CTs, and including the notions of empirical separation and theoretical separation curves, a straightforward reading of the classification rules in the functional field is obtained. Besides, the article offers a series of innovative functional measures and tools to evaluate and interpret the terminal nodes of a functional classification tree. Indeed, a fourth original aim of this article is the presentation of different supplementary criteria for assessing the quality of the leaves and interpret the patterns of FNs, FPs, TNs, and TPs in the context of FCTs. We present different measures such as the FBGSS and FBLSS with this purpose. Ultimately, in addition to the original component related to the interpretation and new functional measures, the results of the functional classifiers FCT and FRF, in terms of accuracy, are excellent. In fact, using one of the two datasets, the FRF based on FPCs breaks the classification world record. In examining the results, we also compare the FRF scheme with a classification based on b‐splines. The latter is not as efficient as the one based on a data‐driven basis. The causes are essentially three. First, a data‐driven basis system better accommodates, by definition, to our data, catching the variability and, hence, the primary information we need. Second, the interpretation of a fixed basis system is aseptic, intricate, and not engaging in considering the time domain. Finally, in terms of accuracy, the performance of the functional classifier based on a fixed basis system is slightly lower than the strategy using a data‐driven basis.

In the application, we have used two datasets regarding ECG data. We are aware of the limitations of this study because the ECG literature is huge and has a long history. To read, analyze, and interpret ECGs, many methods have been proposed in the literature for many decades in the most important international scientific journals, and books.^6^, ^48^, ^49^, ^50^ For this reason, we are conscious that the reading and interpretation of the results for medical purposes requires an accurate interdisciplinary collaboration with experts. Nevertheless, the main goal of this research is to provide methodological tools to aid the interpretation of biomedical signals for professionals who are experts in specific pathologies. Effectively, our method, even if we used ECG signals as an example, can be applied to any biomedical signal, for example, EEG and PFTs. Naturally, this research focuses on medical data, but the methodological approach can be adapted to any time series and also to functions whose domain is different from time. This study shows the application on different datasets not affected by the missing data problem. However, in the functional context, the problem of the existence of some missing data in the time domain does not significantly affect the analysis because the functions and FPCs can still be reconstructed with smoothing techniques. On the other hand, the most severe problem could arise when there are truncated data in the temporal domain, that is, observations that are definitively interrupted at a specific instant of time. There is lively and engaging literature on the problem of performing FPCA in such cases, for example, Shi et al.^51^ Once FPCs have been reconstructed, the proposed approach remains easily implementable.

The proposed line of research, that is, combining FDA and tree‐based supervised classification methods, is appealing and promising. Several potential future extensions and applications may be introduced. The most direct future improvement is to extend this proposal to different types of basis systems such as Fourier or Wavelets basis or create other types of distance or new interpretative tools. From an applicative perspective, as mentioned before, many interesting applications may be done, for example, for monitoring brain activities via EEGs, assessing lung function using pulmonary function tests (PFTs), or studying the different signals adopted to analyze sleep disorders.

## Supporting information

Supporting Information

## Data Availability

Data sharing is not applicable to this article as no new data were created or analyzed in this study.
