# Supplementary material for: Pooling random forest and functional data analysis for biomedical signals supervised classification: Theory and application to electrocardiogram data
Source: Stat Med. 2022 Feb 20;41(12):2247–75. doi: 10.1002/sim.9353 (PMC9303904; doi:10.1002/sim.9353)
Supplement: Supplementary file 1 — Supporting Information [file SIM-41-2247-s001.pdf]

Supplementary material of the paper "*Pooling random forest and functional data analysis for biomedical signals supervised classification: theory and application to electrocardiogram data*"

Abbreviations: FDA, Functional Data Analysis; FPCs, Functional Principal Components; FCTs, Functional Classification Trees; FRF, Functional Random Forest; FBG, Functional Bagging; OOBFD, Out of Bag Functional Data; IBFD, In-Bag Functional Data; ESC, Empirical Splitting Curve; TSC, Theoretical Splitting Curve; FBGSS, Functional Between Groups Sum of Squares; FBLSS, Functional Between Leaves Sum of Squares.

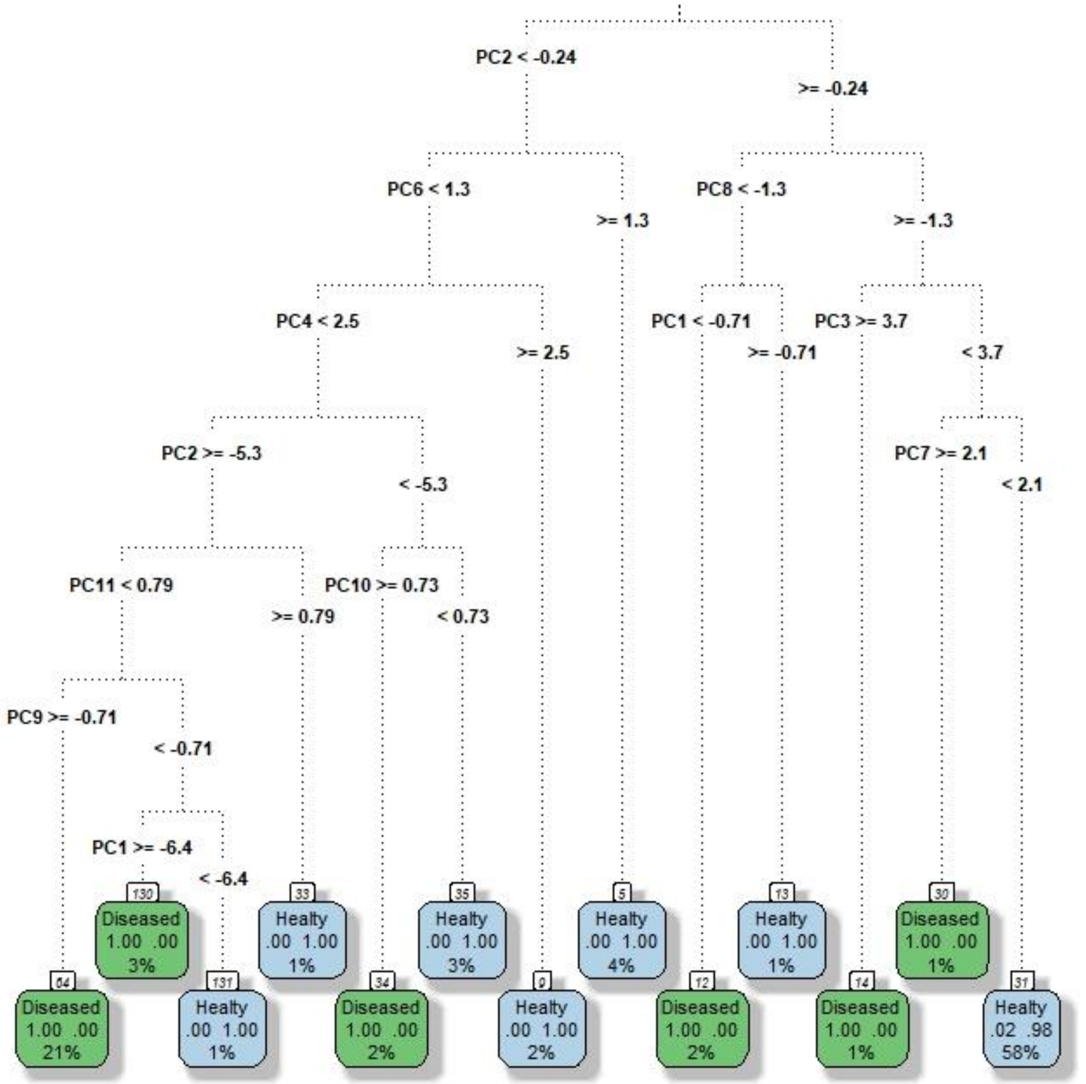

Figure 1. Non-pruned FCT based on the FPCs of the ECG200 dataset. The green leaves indicate a classification as diseased, while the blue leaves lead to the classification of the subjects as healthy. The relative frequencies of subjects of the two groups that make up the terminal leaf are indicated in the boxes. The percentage indicated at the bottom of the box indicates the fraction of people classified following the decision rule that leads to that terminal leaf.

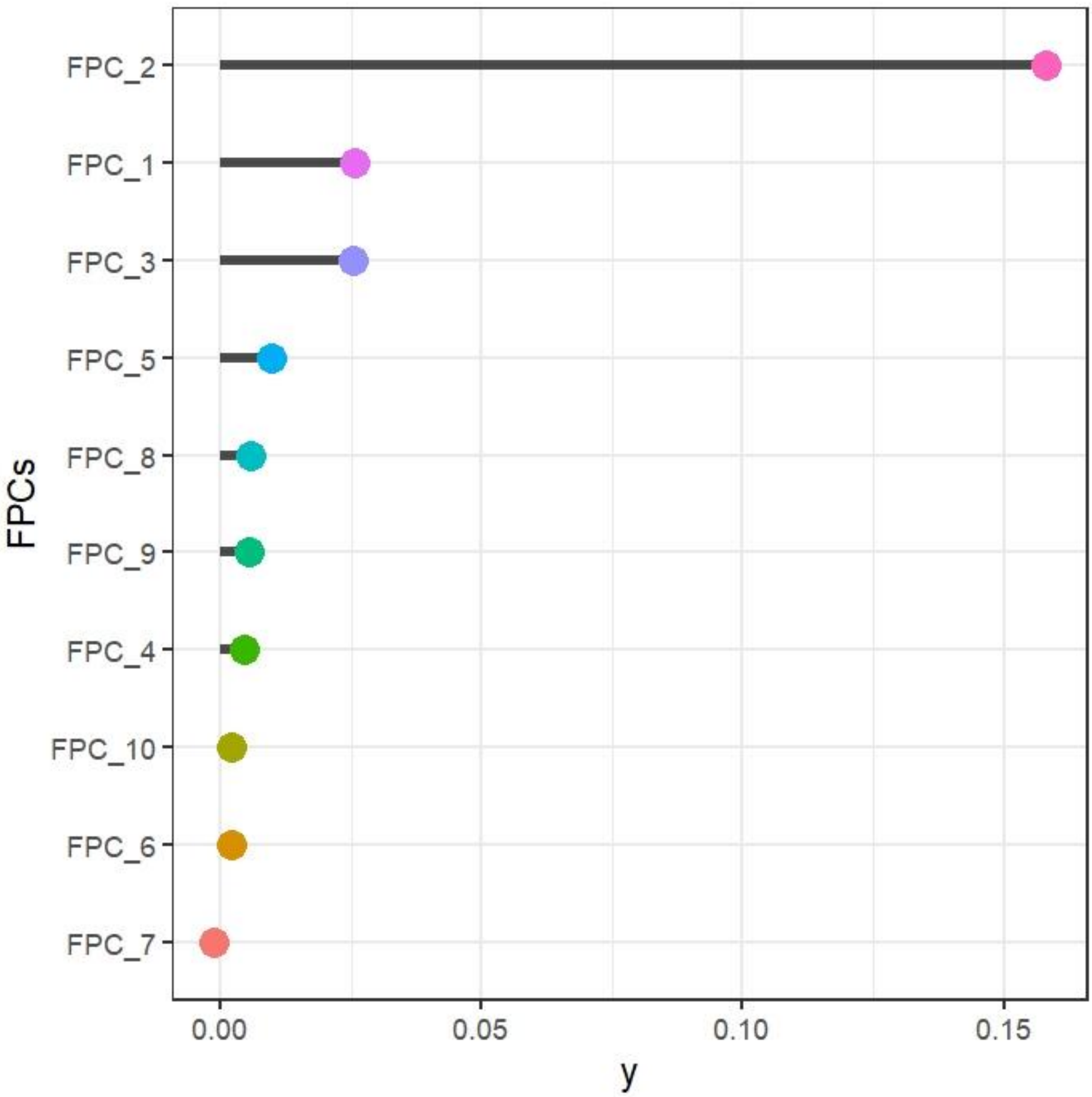

Figure 2. FPCs importance in a single FCT based on FPCs.

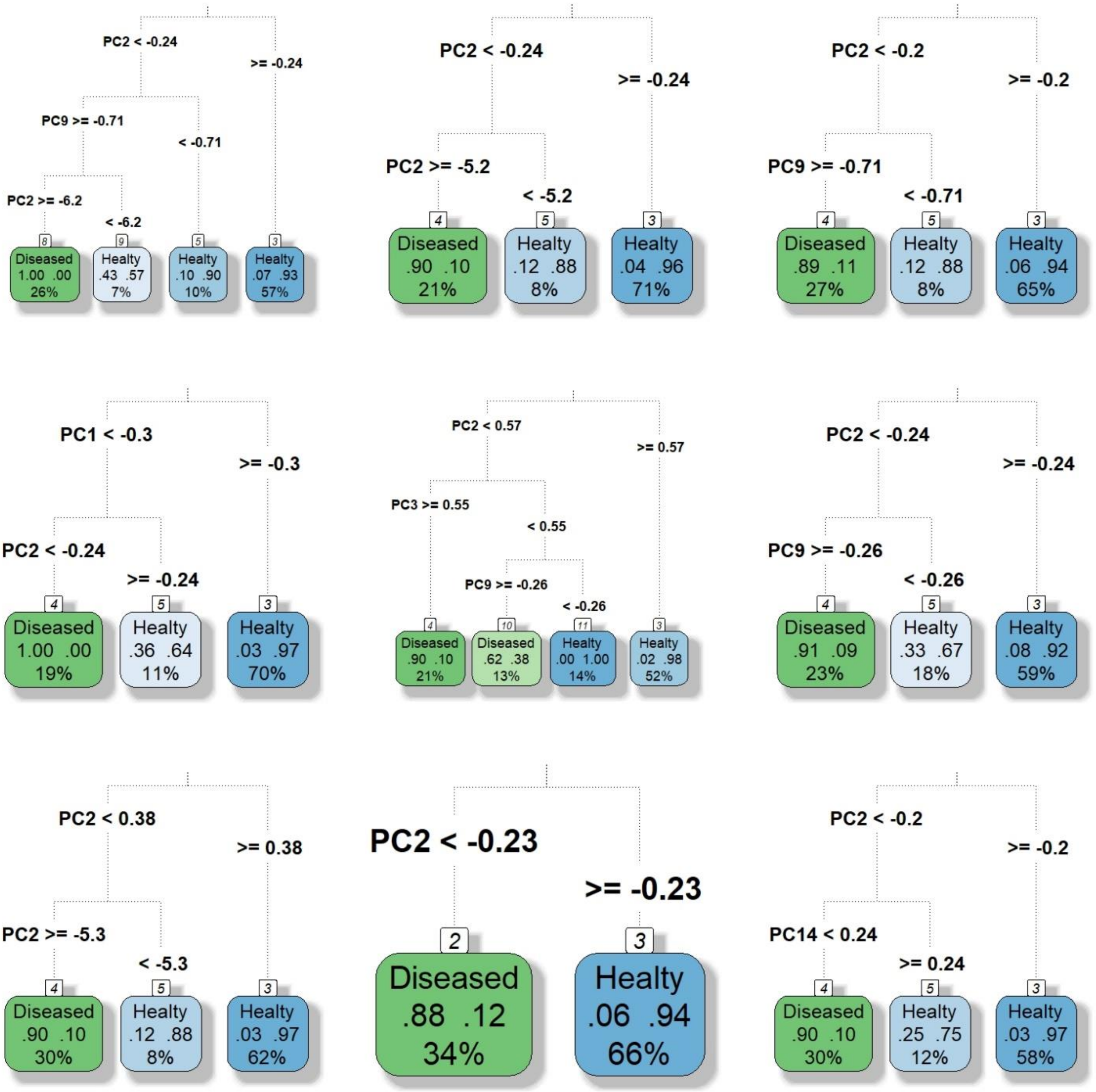

Figure 3. The first nine FCTs obtained using the functional bagging procedure based on the FPCs decomposition. The green leaves indicate a classification as diseased, while the blue leaves lead to the classification of the subjects as healthy. The relative frequencies of subjects of the two groups that make up the terminal leaf are indicated in the boxes. The percentage indicated at the bottom of the box indicates the fraction of people classified following the decision rule that leads to that terminal leaf.

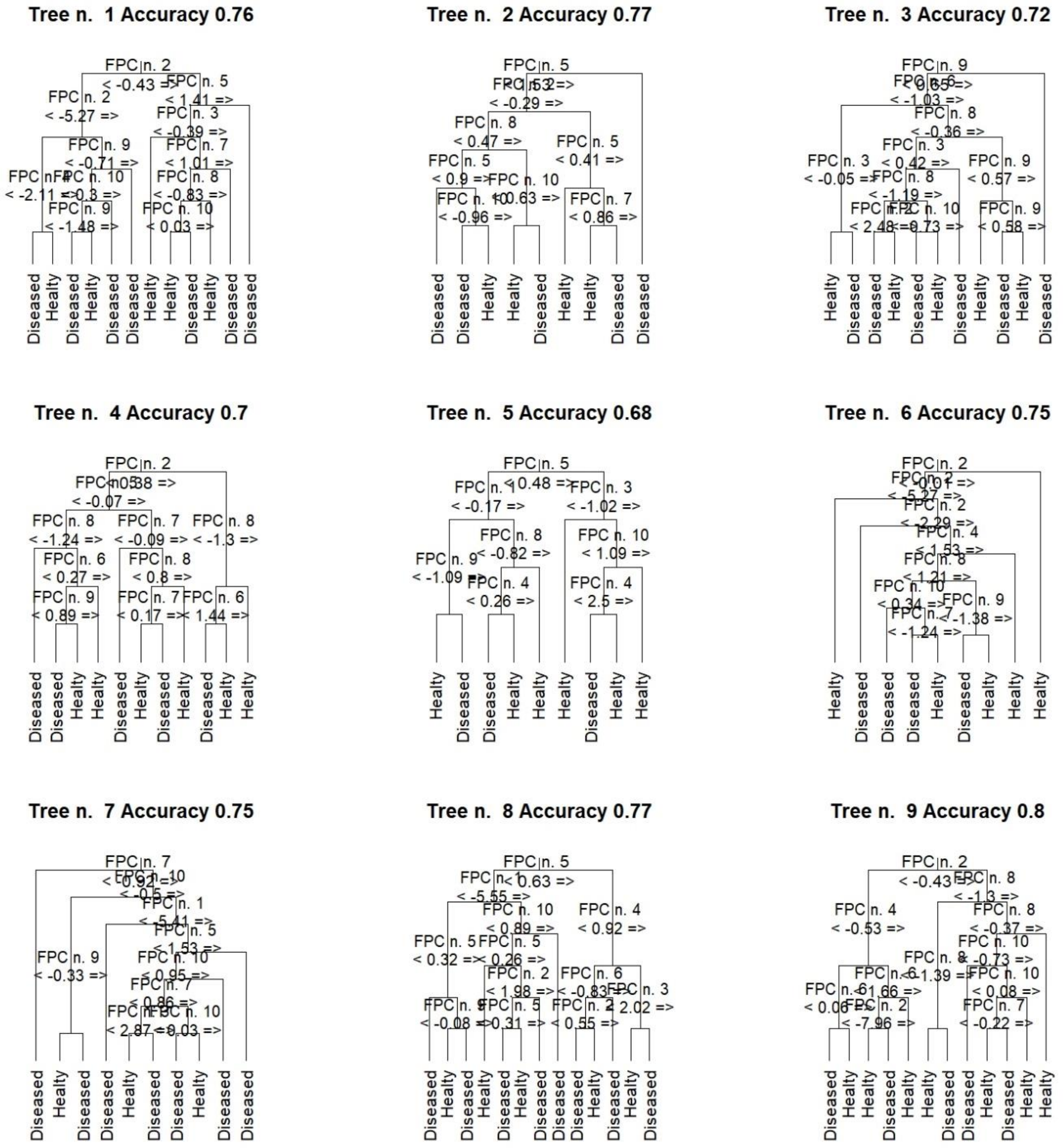

Figure 4. The first nine FCTs obtained using the FRF algorithm based on the FPCs decomposition.

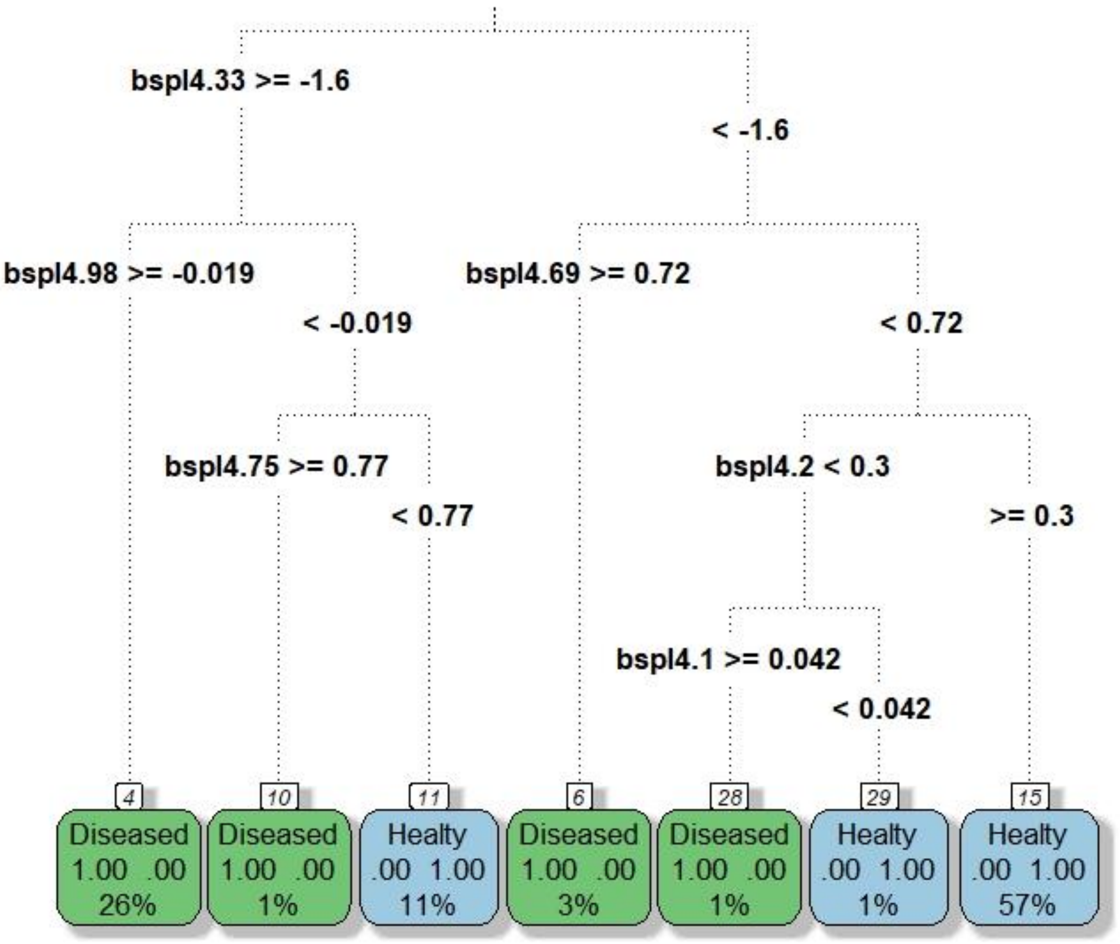

Figure 5. Pruned FRF using b-splines. The green leaves indicate a classification as diseased, while the blue leaves lead to the classification of the subjects as healthy. The relative frequencies of subjects of the two groups that make up the terminal leaf are indicated in the boxes. The percentage indicated at the bottom of the box indicates the fraction of people classified following the decision rule that leads to that terminal leaf.

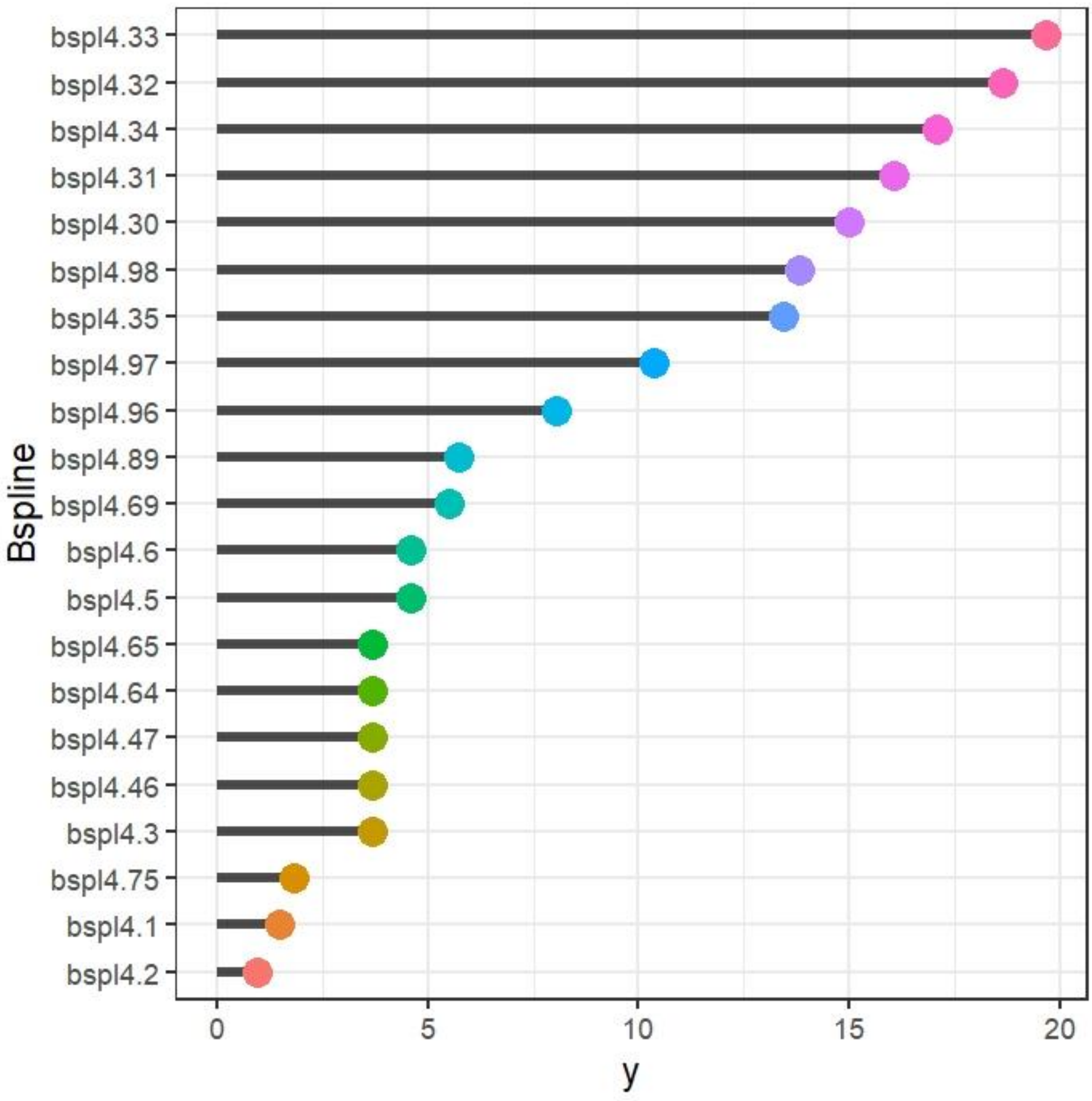

Figure 6. FPCs importance in a single FCT based on b-splines.

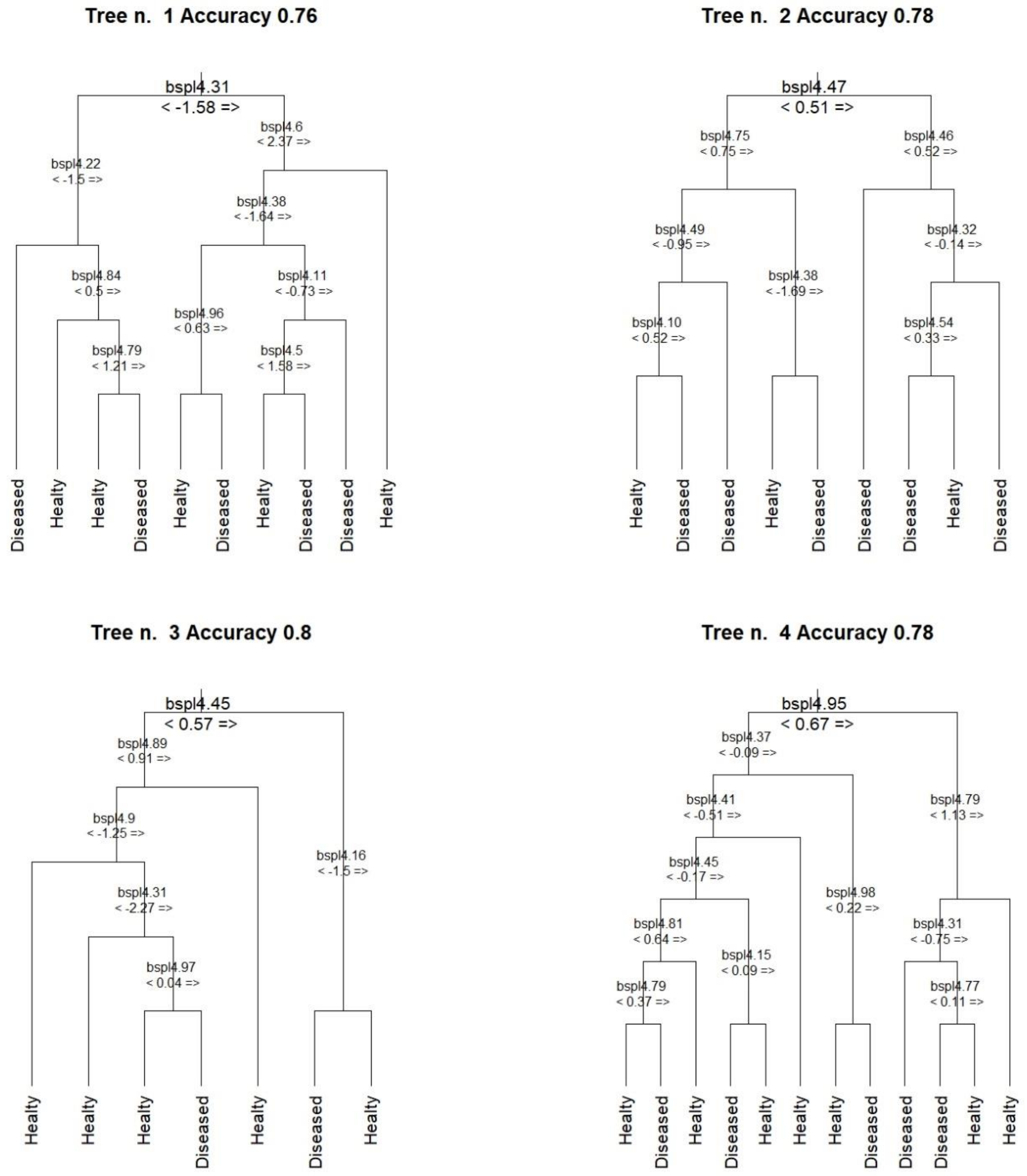

Figure 7. The first four FCTs obtained using the FRF algorithm based on the b-spline decomposition.

	Number of functional principal components involved in the classifier FRF													
N. of Trees	2	3	4	5	6	7	8	9	10	11	12	13	14	15
2	89.38	89.27	89.37	89.87	90.17	90.88	88.18	90.58	87.25	87.23	86.01	87.10	85.23	87.32
3	89.06	88.89	89.66	90.13	90.24	89.09	89.18	88.10	87.25	87.90	86.02	87.57	90.56	89.56
4	90.07	89.79	89.67	90.31	87.71	88.92	90.26	90.82	87.79	90.14	86.30	89.95	89.76	89.17
5	90.48	91.15	91.21	91.90	89.84	90.09	91.24	90.71	90.09	88.77	90.91	89.11	85.52	91.50
6	90.34	89.48	92.24	92.22	92.01	89.63	90.75	92.26	90.11	91.40	89.44	89.96	91.13	90.99
7	90.25	90.17	93.49	91.49	91.68	89.05	91.56	91.56	90.48	87.86	88.84	91.02	90.68	87.50
8	90.89	90.98	91.38	90.49	91.38	90.31	90.80	91.60	91.08	92.42	91.58	89.69	90.78	89.88
9	91.36	90.74	92.51	92.28	92.23	93.12	92.76	91.52	90.71	90.28	92.29	91.06	91.24	90.04
10	91.33	91.50	92.31	92.32	91.52	92.91	92.43	92.74	91.18	91.90	91.94	92.37	90.47	90.71
11	90.95	91.18	90.54	91.52	92.37	91.95	92.34	91.38	91.58	91.00	90.93	91.11	91.58	92.77
12	91.15	92.35	90.78	92.40	91.80	93.95	91.77	92.96	91.97	92.14	92.40	91.77	91.78	91.95
13	91.80	91.35	92.20	92.59	93.17	93.55	92.97	93.19	92.57	91.77	93.19	92.00	92.59	92.38
14	92.00	90.98	93.80	94.00	92.99	93.78	94.78	93.20	93.57	95.20	91.60	93.79	92.59	93.78
15	91.60	91.78	92.37	92.37	93.20	93.59	93.37	93.59	91.58	91.97	93.80	93.59	93.80	93.60
16	91.58	91.40	93.20	92.99	92.38	93.20	93.39	93.00	93.99	93.39	93.17	93.40	93.20	93.20
17	91.80	91.20	92.60	93.80	92.80	93.40	92.79	92.80	93.00	93.79	92.20	93.20	92.40	92.20
18	92.00	93.20	92.80	94.60	92.60	93.80	93.00	93.60	92.60	93.20	92.80	93.00	92.60	92.80
19	93.00	91.18	93.40	93.20	92.60	93.40	93.40	93.40	92.40	94.00	94.00	93.60	94.60	93.60
20	92.20	91.58	92.80	94.20	91.98	93.80	93.60	93.20	93.40	93.40	92.59	93.80	93.60	92.18
21	92.80	92.20	93.60	92.20	93.60	93.60	93.20	93.40	94.00	94.60	94.00	93.20	93.00	93.60
22	92.20	92.40	92.80	93.40	94.20	94.00	93.20	93.80	94.60	93.80	93.20	93.40	94.00	94.00
23	91.60	92.60	93.80	94.20	93.40	93.00	93.40	94.20	92.60	94.20	93.40	93.80	93.40	94.00
24	92.00	92.80	93.40	93.80	93.20	93.40	93.00	93.00	93.60	93.60	93.60	93.40	93.60	93.40
25	91.80	92.40	93.20	94.40	93.80	93.20	93.80	94.20	93.40	93.20	94.20	93.40	94.00	93.60
26	92.60	91.80	94.00	93.80	93.40	93.60	93.60	93.60	94.00	93.40	93.80	94.40	93.60	93.60
27	92.20	92.40	93.00	93.00	94.00	93.80	93.60	94.20	94.00	94.80	93.60	93.20	92.60	94.40
28	92.00	91.80	93.60	94.00	94.20	94.40	93.60	93.80	94.00	93.80	94.20	94.00	94.00	93.20
29	92.60	92.60	93.60	93.40	94.20	93.20	94.00	94.00	93.60	94.40	93.40	94.00	94.20	94.20
30	91.60	93.00	93.80	93.80	93.80	94.40	93.60	93.40	93.60	94.00	94.20	93.60	94.80	94.20
31	92.00	92.60	93.00	93.40	94.20	94.40	93.40	94.20	93.60	94.00	93.60	94.80	93.80	93.60
32	92.00	91.80	92.80	93.40	94.00	94.20	94.00	93.40	94.60	94.40	95.00	93.60	93.20	93.60
33	92.00	93.20	93.60	94.00	94.20	93.20	93.20	94.20	93.60	93.60	94.00	94.40	93.80	94.00
34	91.60	92.80	94.00	93.80	93.40	93.60	93.00	94.00	94.60	94.00	93.80	93.40	93.20	93.80
35	92.20	92.60	93.40	94.00	94.20	93.80	94.20	94.00	93.80	93.20	94.00	94.00	93.80	93.80
36	92.00	92.80	93.20	93.60	94.00	94.40	93.40	94.00	94.60	94.40	94.00	94.60	93.60	94.00
37	92.00	92.40	94.00	93.20	93.80	94.20	94.40	93.40	93.80	94.40	93.80	93.40	93.60	94.20
38	92.00	92.60	93.80	94.00	93.40	93.80	93.40	94.60	94.20	93.80	94.20	94.40	94.40	94.60
39	92.20	92.40	93.80	93.20	92.80	93.80	94.60	94.20	94.00	94.40	94.00	93.20	94.60	93.40
40	92.40	92.60	93.40	93.40	94.40	93.80	94.20	94.20	93.20	93.20	93.60	94.00	93.60	94.20
45	92.40	93.00	93.80	94.20	94.40	93.60	94.00	93.60	94.00	94.00	94.20	94.40	93.80	94.00
50	92.40	93.20	93.80	93.80	94.20	94.00	94.00	93.80	93.40	94.40	94.40	94.20	93.80	94.40

TABLE 1 Accuracy for the ECG5000 dataset using the training set: Accuracy of the FRF classifier according to different sizes of the forest and different numbers of FPCs. The best accuracy on the training set is 97.28% with 4 FPCs and 20 trees.

	Number of functional principal components involved in the classifier FRF													
N. of Trees	2	3	4	5	6	7	8	9	10	11	12	13	14	15
2	89.22	90.07	90.27	88.76	88.64	88.98	89.47	88.73	89.11	88.67	87.58	90.00	88.36	89.56
3	90.29	91.42	91.22	90.96	91.73	91.16	91.58	91.71	90.51	90.93	90.07	89.96	91.40	91.76
4	91.24	90.13	91.56	91.64	92.13	91.40	91.91	91.18	91.07	91.53	91.89	90.82	91.60	90.38
5	91.56	91.22	91.96	92.38	92.49	92.36	92.16	92.00	92.42	92.27	92.11	91.18	91.51	91.89
6	90.80	91.27	91.69	92.07	91.89	92.44	91.84	92.73	91.82	92.40	92.89	92.42	91.53	92.00
7	91.02	91.53	92.67	92.29	92.91	92.73	92.89	91.69	91.82	91.42	92.18	92.64	91.80	92.20
8	90.91	92.07	92.56	92.80	92.58	92.38	92.51	91.93	92.78	92.58	92.22	92.64	93.02	92.53
9	91.87	91.58	92.49	92.67	93.00	93.02	91.98	92.80	92.29	91.78	92.82	92.47	92.76	92.51
10	91.73	92.13	92.42	92.42	92.82	93.07	92.64	92.42	92.33	92.60	92.38	92.51	92.82	92.56
11	92.04	91.82	92.33	92.91	92.56	93.24	92.29	92.62	93.02	92.89	92.38	93.04	92.87	92.96
12	91.67	91.47	91.87	92.71	92.98	92.76	92.93	93.40	93.11	93.20	92.60	92.67	92.04	92.89
13	91.53	92.22	92.53	92.82	92.84	93.16	93.13	92.76	92.29	92.91	92.98	92.78	93.00	92.64
14	91.71	91.93	92.51	93.07	92.91	92.62	92.98	92.89	92.98	93.11	92.91	92.16	92.93	92.76
15	90.96	91.87	92.47	92.71	92.84	92.89	93.02	92.76	93.11	92.87	93.49	92.78	93.47	93.36
16	92.09	92.16	92.07	92.76	92.87	92.71	92.87	92.89	92.13	92.91	93.00	92.02	92.91	93.00
17	91.62	91.93	92.62	92.33	92.91	93.13	93.13	93.22	92.89	92.89	93.16	92.42	93.13	93.09
18	91.67	92.11	92.51	92.80	93.07	93.24	93.16	93.20	92.67	93.16	92.96	93.04	92.60	93.09
19	91.87	92.44	92.40	92.91	92.78	93.18	92.96	93.04	92.87	92.91	93.29	93.36	92.69	92.47
20	91.76	91.96	92.93	92.73	93.11	93.02	93.04	93.09	92.71	92.78	93.18	92.76	92.89	92.91
21	91.96	92.22	92.56	93.29	92.89	93.38	93.04	93.13	92.73	92.87	92.96	92.96	93.22	92.78
22	91.71	91.89	92.73	92.93	92.93	93.20	92.87	93.04	92.71	93.11	93.27	92.82	93.16	92.93
23	92.04	92.36	92.84	92.73	93.18	93.16	93.24	92.60	92.78	92.84	93.18	92.89	92.98	93.09
24	91.78	91.96	92.96	92.89	93.22	92.71	93.09	93.36	92.96	93.09	92.98	93.00	93.47	93.18
25	91.80	92.13	92.58	93.16	92.96	92.93	93.44	92.87	93.16	93.24	93.42	93.00	93.07	93.29
26	91.78	92.16	92.51	93.00	93.13	93.13	92.78	93.13	93.09	93.24	93.22	93.00	92.84	93.07
27	92.16	91.91	92.60	93.07	93.18	92.89	93.24	93.27	93.27	93.31	93.38	92.36	93.24	92.98
28	92.07	92.20	92.51	93.04	93.18	92.98	93.07	93.27	93.11	93.09	93.07	92.98	93.42	92.96
29	91.87	92.02	92.60	92.91	92.80	93.07	92.80	93.27	92.80	93.31	92.87	93.07	92.91	93.07
30	91.89	92.31	92.42	92.98	92.80	92.96	93.07	93.44	93.00	93.11	93.20	93.13	93.04	92.91
31	92.13	92.20	92.82	93.11	93.18	92.98	93.33	93.00	93.09	93.24	93.00	92.82	93.09	93.22
32	91.76	92.18	92.76	92.80	93.27	93.07	92.93	93.27	93.27	93.18	93.09	93.02	93.20	92.84
33	91.98	92.11	92.96	92.78	93.38	93.00	93.22	92.71	92.78	92.82	93.29	93.16	93.04	93.29
34	92.16	91.82	92.56	92.89	92.87	93.38	92.69	93.18	93.22	92.64	92.98	92.91	93.27	93.18
35	91.87	91.87	93.04	93.00	93.29	92.91	93.07	92.96	93.27	93.07	92.71	93.11	93.51	93.11
36	91.89	92.29	92.49	93.16	93.16	93.20	93.22	93.00	93.27	92.91	93.13	92.98	93.31	92.84
37	91.78	92.00	92.51	93.18	93.18	93.18	92.98	93.20	93.18	93.13	93.11	93.00	93.24	92.93
38	91.73	92.13	93.02	93.04	93.07	92.91	93.16	93.33	93.09	92.69	93.16	92.98	93.22	93.22
39	92.00	92.00	92.73	93.36	93.31	93.20	93.24	93.36	93.18	93.13	93.29	92.93	93.24	93.51
40	92.11	92.07	92.53	93.24	93.64	93.22	93.16	93.11	93.02	93.16	93.18	92.76	93.18	93.22
45	91.80	92.11	92.96	92.93	93.11	93.24	92.93	92.96	93.16	92.69	93.29	92.93	93.51	93.16
50	91.82	92.09	92.89	93.07	93.18	93.00	93.29	93.20	93.11	93.04	93.27	93.24	93.02	92.91

TABLE 2 Accuracy for the ECG5000 dataset using the test set: Accuracy of the FRF classifier according to different sizes of the forest and different numbers of FPCs. The best accuracy on the test set is 93.64% with 6 FPCs and 40 trees. The world top accuracy reached for this dataset is still 94.61% with the COTE algorithm. https://www.timeseriesclassification.com/description.php?Dataset=ECG5000

N. of Trees	Accuracy on Training Set	Accuracy on Test Set
2	89.25	89.49
3	88.35	91.87
4	88.32	92.24
5	91.84	93.18
6	91.54	92.20
7	93.21	93.29
8	90.89	92.82
9	91.43	93.22
10	92.77	93.00
11	92.54	93.29
12	92.20	92.80
13	92.99	93.27
14	92.38	93.38
15	94.40	93.38
16	93.19	93.24
17	93.00	93.53
18	94.00	93.31
19	93.60	93.49
20	93.80	93.29
21	94.40	93.84
22	94.60	93.62
23	95.40	93.38
24	94.60	93.87
25	94.00	93.60
26	94.20	93.44
27	93.80	93.38
28	94.40	93.67
29	95.20	93.51
30	94.80	93.53
31	94.80	93.49
32	94.80	93.49
33	94.60	93.47
34	94.60	93.67
35	94.40	93.47
36	94.80	93.36
37	93.80	93.69
38	93.60	93.58
39	94.80	93.49
40	95.20	93.62
45	94.60	93.62
50	94.80	93.58

TABLE 3 Accuracy for the ECG5000: Accuracy of the FRF-B-spline classifier according to different sizes of the forest and a fixed number of B-splines. The best accuracy on the training set is 95.20% with 29 trees. The best accuracy on the test set is 93.87% with 24 trees.

N. of NNs in functional KNN	Accuracy on Training Set	Accuracy on Test Set
1	93.60	92.31
3	94.60	93.42
5	94.80	93.68
7	94.80	93.62
9	95.00	93.64
11	93.20	93.44
13	93.00	92.69
15	93.00	92.04
17	93.00	92.09
19	93.00	92.09
21	93.00	92.07
23	93.00	92.02
25	93.00	92.02

TABLE 4 Accuracy for the ECG5000 dataset: Functional classification using the k-nn classifier of the R package fda.usc. The best accuracy on training set is 95.00% with 9 nearest neighbours. The best accuracy on the test set is 93.64% with 9 nearest neighbours.

Functional Depth Measure	Accuracy on the Training Set	Accuracy on the Test Set
	Train	Test
RP	61.60	63.16
mode	93.60	91.87
RT	77.60	73.27
FM	65.20	68.04
RPD	65.20	68.04

TABLE 5 Accuracy for the ECG5000: Functional classification using depth classifiers of the R package fda.usc. The best accuracy on the training set is 93.60% with the depth measure “mode”. The best accuracy on the test set is 91.87% with the depth measure “mode”. depth.RP computes the Random Projection depth (see Cuevas et al. 2007). depth.mode implements the modal depth (see Cuevas et al 2007). depth.RT implements the Random Tukey depth (see Cuesta–Albertos and Nieto–Reyes 2008). depth.FM computes the integration of an univariate depth along the axis x (see Fraiman and Muniz 2001). It is also known as Integrated Depth. depth.RPD implements a depth measure based on random projections possibly using several derivatives (see Cuevas et al. 2007).
